# *AcademH*, a lineage of *Academ* DNA transposons encoding helicase found in animals and fungi

**DOI:** 10.1186/s13100-020-00211-1

**Published:** 2020-04-18

**Authors:** Kenji K. Kojima

**Affiliations:** Genetic Information Research Institute, Cupertino, CA 95014 USA

**Keywords:** DNA transposon, *Academ*, Helicase, Target site duplication, Transposase, Plant homeodomain

## Abstract

**Background:**

DNA transposons are ubiquitous components of eukaryotic genomes. *Academ* superfamily of DNA transposons is one of the least characterized DNA transposon superfamilies in eukaryotes. DNA transposons belonging to the *Academ* superfamily have been reported from various animals, one red algal species *Chondrus crispus*, and one fungal species *Puccinia graminis*. Six *Academ* families from *P. graminis* encode a helicase in addition to putative transposase, while some other families encode a single protein which contains a putative transposase and an XPG nuclease.

**Results:**

Systematic searches on Repbase and BLAST searches against publicly available genome sequences revealed that several species of fungi and animals contain multiple *Academ* transposon families encoding a helicase. These *AcademH* families generate 9 or 10-bp target site duplications (TSDs) while *Academ* families lacking helicase generate 3 or 4-bp TSDs. Phylogenetic analysis clearly shows two lineages inside of *Academ*, designated here as *AcademH* and *AcademX* for encoding helicase or XPG nuclease, respectively. One sublineage of *AcademH* in animals encodes plant homeodomain (PHD) finger in its transposase, and its remnants are found in several fish genomes.

**Conclusions:**

The *AcademH* lineage of TEs is widely distributed in animals and fungi, and originated early in the evolution of *Academ* DNA transposons. This analysis highlights the structural diversity in one less studied superfamily of eukaryotic DNA transposons.

## Introduction

Transposable elements (TEs), or transposons are ubiquitous components of genomes in all three domains of life [1, 2]. TEs are traditionally classified into 2 classes: Class I retrotransposons and Class II DNA transposons [3]. Autonomous retrotransposons encode a reverse transcriptase and during the transposition, the information of RNA is transformed into DNA by reverse transcription. DNA transposons do not have a process of reverse transcription in their transposition cycle. At least 5 independent DNA-cleaving/recombining enzymes (DDE transposase or DDD/E transposase, tyrosine recombinase, serine recombinase, HUH nuclease, and Cas1 endonuclease) have been incorporated into TEs and related mobile genetic elements [4, 5]. DDE transposase or integrase is the most ubiquitous enzyme that functions as transposase of DNA transposons, as well as of long terminal repeat (LTR) retrotransposons and of retroviruses [6]. Eukaryotic DNA transposons are now classified into around 20 superfamilies [1]. Most of these superfamilies, such as *Mariner/Tc1* and *Harbinger/PIF1*, are known to encode a DDE transposase.

DDE transposase is topologically a member of RNaseH-like fold [6]. The conserved core of the transposase domain is β1-β2-β3-α1-β4-α2/3-β5-α4-α5. Three acidic residues, DDD or DDE play the central role in the transposition. The first D is located on β1 and the second D on or just after β4. The last D or E is on or just before α4. In the case of the integrase encoded by human immunodeficiency virus type 1 (HIV-1), the distance between the second D and the last E is 35 residues. In some DNA transposons, the catalytic core domain between β5 and α4 is extended by “insertion domain.” In the case of RAG1, recombination activating gene 1, which originated from an eukaryotic DNA transposon superfamily *Transib* [7], the insertion domain is 264 residues in length and entirely α-helical [6]. The transposase encoded by *Hermes*, a member of eukaryotic DNA transposon superfamily *hAT*, contains a 288-aa-long insertion domain [6].

**Fig. 1:**
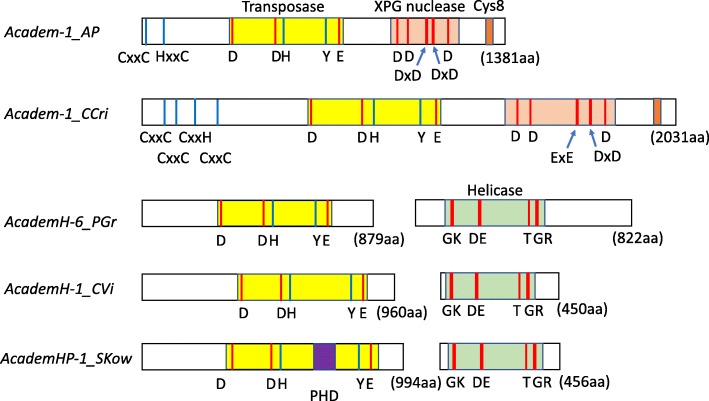
Schematic structures of proteins encoded by *Academ* transposons. Encoded proteins are shown as open boxes, and inside them, protein domains are shown as colored boxes. Conserved residues are shown as bars in red and blue. Protein lengths are shown in parentheses. Transposase and helicase proteins of *AcademH* families are encoded in opposite directions

The *Academ* superfamily of eukaryotic DNA transposons was first described by Kapitonov and Jurka [8] from various animals. To date, *Academ* has been found from animals, fungi, and plants [1]. In animals, *Academ* is widely distributed and found from genomes of 7 phyla: Chordata, Hemichordata, Echinodermata, Annelida, Mollusca, Arthropoda, and Cnidaria. In contrast, in fungi and in plants, only one species of each group is reported to have *Academ* transposons: a red alga *Chondrus crispus* [9] and a pathogenic fungus *Puccinia graminis* [10], while the wide distribution of *Academ* in fungi was suggested [11]. The transposase domain of *Academ* is predicted to be a DDE transposase [12]. An entirely α-helical insertion domain was predicted between β5 and α4, as are the cases of RAG1 and *Hermes*. Another insertion domain was predicted between β2 and β3, unlike any other transposases. Many of *Academ* families encode a large protein that contains three recognizable domains, a transposase, an XPG nuclease, and a putative Cys8 zinc finger [8] (Fig. 1). These three domains can be recognized among *Academ* families from animals and *C. crispus*. The *Academ* families from *P. graminis* do not encode an XPG nuclease. Instead, they encode a superfamily II helicase as a separate protein [10] (Fig. 1). This lineage was designated as *AcademH*. It is not yet known whether the presence of helicase is a recently acquired characteristic specific for *Academ* families from *P. graminis*, or it is an ancient trait shared by various *Academ* families from diverse organisms.

In this study, many families of *AcademH* from other fungi and animals were characterized. No intact *AcademH* transposons were found from vertebrates, but some fish genomes still contain remnants of *AcademH* transposons. *AcademH* shows 9 or 10-bp target site duplications (TSDs), although other *Academ* shows 3 or 4-bp TSDs. The sequence comparison and phylogenetic analysis revealed two independent lineages with different TSD length and protein composition inside of the *Academ* DNA transposons.

## Results

### *Academ* families encoding a superfamily II helicase

Manual inspection of Repbase entries revealed that besides 6 *AcademH* families from *P. graminis*, *Academ-1_ADi*, *Academ-2_ADi*, *Academ-3_ADi* from the coral *Acropora digitifera*, and *Academ-2_CGi* from the Pacific oyster *Crassostrea gigas* also encode a superfamily II helicase protein. Using the helicase protein sequences from these families as queries, Censor search [13] against published genome sequences was performed. It led to the characterization of *AcademH* families encoding a helicase protein from 7 species of basidiomycetes fungi (*Laccaria bicolor, Puccinia coronata*, *Puccinia horiana*, *Puccinia striiformis*, *Puccinia triticina*, *Serpula lacrymans*), one species of fungi in Mucoromycotina (*Lobosporangium transversale*), and another oyster *Crassostrea virginica*, in addition to more families from the three species above (Table 1 and Supplementary Dataset S1). Non-autonomous DNA transposons showing similarity in terminal regions with *AcademH* families were also found from three cnidarians (*Exaiptasia pallida*, *Orbicella faveolata*, and *Stylophora pistillata*) and the Yesso scallop *Mizuhopecten yessoensis* (Table 1 and Supplementary Dataset S1).

**Table 1:** *AcademH* distribution

Classification	Organism	***AcademH*** families
Fungi/Basidiomycota		
-Pucciniomycetes/Pucciniales	*Puccinia coronata*	*AcademH-1_PCor* to *17_PCor*, *AcademH-14N1_PCor*, *AcademH-N1_PCor* to *N13_PCor*
	*Puccinia graminis*	*AcademH-1_PG* to *6_PG*, *AcademH-N2_PGr* to *N6_PGr, AcademH-2B_PGr, AcademH-N3B_PGr*
	*Puccinia horiana*	*AcademH-1_PHor*, *AcademH-N1_PHor* to *N25_PHor*
	*Puccinia sorghi*	*AcademH-N1_PSor*
	*Puccinia striiformis*	*AcademH-1_PSt* to *18_PSt*, *AcademH-N1_PSt* to *N12_PSt*
	*Puccinia triticina 1–1 BBBD Race 1*	*AcademH-1_PTrit* to *6_PTrit*, *AcademH-1B_PTrit*, *AcademH-N1_PTrit* to *N20_PTrit*
	*Melampsora larici-populina*	*AcademH-N1_MLP*
-Agaricomycetes/Auriculariales	*Exidia glandulosa HHB12029*	*AcademH-1_ExGl*
-Agaricomycetes/Atheliales	*Fibularhizoctonia sp. CBS 109695*	*AcademH-1_FiCBS*
-Agaricomycetes/Agaricales	*Gymnopilus dilepis*	*AcademH-1_GyDi*
	*Hebeloma cylindrosporum h7*	*AcademH-1_HeCy*
	*Laccaria bicolor S238N-H82*	*AcademH-1_LB*, *AcademH-2_LB*
	*Panaeolus cyanescens*	*AcademH-1_PaCy*, *AcademH-2_PaCy*
-Agaricomycetes/Amylocorticiales	*Plicaturopsis crispa FD-325 SS-3*	*AcademH-1_PlCr* to *3_PlCr*
-Agaricomycetes/Boletales	*Serpula lacrymans*	*AcademH-1_SLL*
-Agaricomycetes/Hymenochaetales	*Schizopora paradoxa*	*AcademH-1_ScPa*, *AcademH-2_ScPa*
-Agaricomycetes/Polyporales	*Dichomitus squalens LYAD-421 SS1*	*AcademH-1_DiSq*
	*Trametes cinnabarina*	*AcademH-1_TrCi*, *AcademH-2_TrCi*
	*Trametes pubescens*	*AcademH-1_TrPu*
-Exobasidiomycetes/Tilletiales	*Tilletia caries*	*AcademH-1_TiCa*, *AcademH-2_TiCa*
	*Tilletia indica*	*AcademH-1_TiIn*
Fungi/Ascomycota		
-Pezizomycetes/Pezizales	*Ascobolus immersus RN42*	*AcademH-1_AsIm*, *AcademH-2_AsIm*
Fungi/Mucoromycota		
-Mortierellomycetes/Mortierellales	*Lobosporangium transversale*	*AcademH-1_LoTr*
	*Mortierella verticillata NRRL 6337*	*AcademH-1_MoVe*, *AcademH-2_MoVe*
Metazoa/Porifera		
-Demospongiae/Haplosclerida	*Amphimedon queenslandica*	*AcademH-1_AQ* to *3_AQ*, *AcademH-N1_AQ* to *N2_AQ*
Metazoa/Cnidaria		
-Anthozoa/Scleractinia	*Acropora digitifera*	*Academ-1_ADi* to *3_ADi*, *AcademH-4_ADi* to *7_ADi*, *AcademH-N1_ADi* to *N14_ADi*
	*Orbicella faveolata*	*AcademH-N1_OrFa* to *N6_OrFa*
	*Stylophora pistillata*	*AcademH-1_StPi*, *AcademH-N1_StPi* to *N3_StPi*
-Anthozoa/Actiniaria	*Exaiptasia pallida*	*AcademH-1_ExPa* to *2_ExPa*, *AcademH-N1_ExPa* to *N3_ExPa*
	*Nematostella vectensis*	*AcademH-N1_NV*, *N1A_NV*, *N2_NV*, *N2A_NV*
Metazoa/Priapulida		
-Priapulimorpha/Priapulimorphida	*Priapulus caudatus*	*AcademHP-1_PrCa*
Metazoa/Mollusca		
-Bivalva/Ostreoida	*Crassostrea gigas*	*Academ-2_CGi, AcademH-1_CGi* to *3_CGi*, *AcademH-2N1_CGi, AcademH-N1_CGi to N3_CGi*, *AcademH-2B_CGi*
	*Crassostrea virginica*	*AcademH-1_CVi* to *16_CVi*, *AcademH-2N1_CVi*, *AcademH-7N1_CVi*, *AcademH-N1_CVi* to *N2_CVi*, *AcademHP-1_CVi*
-Bivalva/Pectinoida	*Mizuhopecten yessoensis*	*AcademH-N1_MiYe* to *N3_MiYe*
Metazoa/Hemichordata		
-Euteropneusta	*Saccoglossus kowalevskii*	*AcademHP-1_SKow* to *2_SKow*, *AcademH-N1_SKow*, *AcademH-N1B_SKow*
Metazoa/Echinodermata		
-Echinoidea/Echinoida	*Strongylocentrotus purpuratus*	*AcademHP-1_SP*
Metazoa/Chordata		
-Leptocardii/Amphioxiformes	*Branchiostoma floridae*	*AcademH-N1_BF*
	*Branchiostoma belcheri*	*AcademH-1_BBe*, *AcademH-2_BBe*

With two of these characterized *AcademH* protein sequences (*AcademH-6_PGr* and *AcademH-1_CVi*) as queries, BLASTP search against the non-redundant protein sequences (nr) at NCBI BLAST website hits many proteins from diverse fungi and animals (Supplementary Table S1). In fungi, proteins related to *AcademH* transposases were found from three subdivisions (Agaricomicotina, Pucciniomycotina, Ustilaginomycotina) within Basidiomycota, one subdivision (Pezisomycotina) within Ascomycota, and one subdivision (Mortierellomycotina) within Mucoromycota. Despite the report that *Academ* transposons are widely distributed in fungi [11], no other fungal group was revealed to contain *AcademH* transposons in this analysis. In animals, genomes from 9 phyla (Porifera, Cnidaria, Mollusca, Annelida, Brachiopoda, Priapulida, Chordata, Hemichordata, and Echinodermata) encode proteins related to *AcademH* transposases. Most of these protein sequences were encoded by single-copy, non-repetitive sequences. Basidiomycetes fungi with more than 5 protein hits and all other species were further analyzed. If their terminal inverted repeats (TIRs) longer than 10 bp and TSDs adjacent to TIRs could be detected in flanking 10,000-bp sequences, they were considered as full-length *Academ* transposons (Table 1 and Supplementary Dataset S2). Most of these single-copy *Academ* transposons encode a helicase protein. The sequence lengths, numbers of uninterrupted full-length copies, and the sequence identities to the consensus sequences are shown in Supplementary Table S2.

**Fig. 2:**
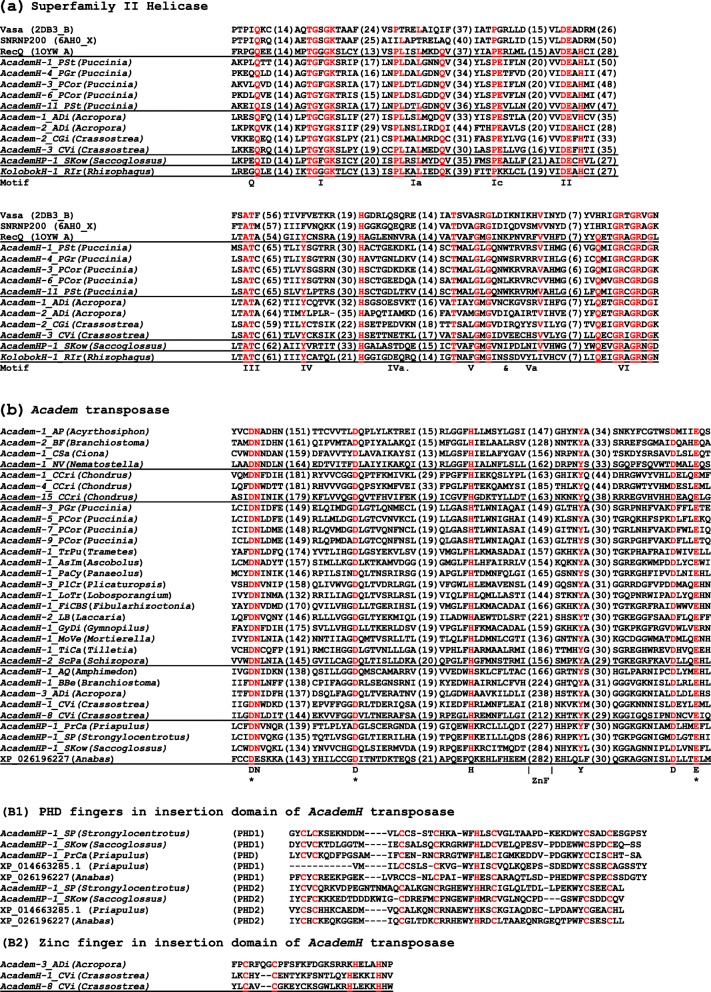
Motifs of protein domains conserved in the *Academ* superfamily. Conserved residues are shown in red. The lengths between motifs are shown in parentheses. **a** Superfamily II helicase. The motif names are shown below alignment. **b***Academ* transposase. Conserved residues are shown below alignment. Asterisks indicate three catalytic residues. ZnF indicates the location where animal transposases encode PHD fingers or another zinc finger motif, which are shown in (B1) and (B2), respectively

Secondary structure-based protein homology search HHpred was performed with helicase proteins encoded by *AcademH* DNA transposons. The top hit was RecQ DNA helicase from *Escherichia coli*, followed by U5 small nuclear ribonucleoprotein 200 and RNA helicase Vasa. The pairwise alignment generated by HHpred and multiple protein alignment generated by MAFFT were combined. It revealed that *AcademH* helicases conserve all motifs important for catalytic reactions, nucleic acid binding, and ATP binding (Fig. 2a). Censor search using helicase proteins encoded by *AcademH* against Repbase hit some families of *KolobokH*, a lineage of *Kolobok* DNA transposons encoding a helicase [14]. However, helicases encoded by *AcademH* and *KolobokH* are not so closely related to each other and are likely acquired independently in these two lineages of DNA transposons (data not shown). Helicases encoded by *Helitron* DNA transposons are Superfamily I helicases related to PIF1 helicase [15], and thus, there is little sequence similarity between helicases encoded by *AcademH* and *Helitron*.

*Academ* families without helicase often, but not always, contain 1 long open reading frame for a large protein containing three recognizable domains: a transposase, an XPG nuclease, and a putative Cys8 zinc finger (Figs. 1 and 2). Here, *Academ* families with XPG nuclease are designated as *AcademX*. In contrast, *AcademH* usually contain introns and encodes two proteins in opposite directions. These two proteins are encoded without overlapping. None of *AcademH* families encode an XPG nuclease or a Cys8 zinc finger.

### Longer TSDs generated by *AcademH* than *AcademX* families

**Fig. 3:**
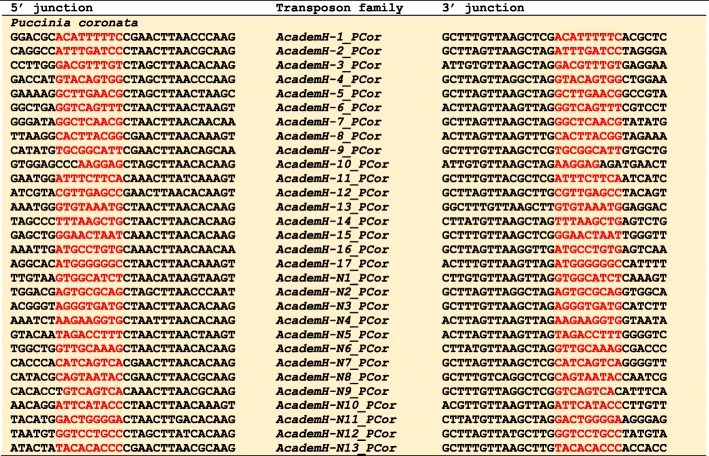
Termini and TSDs of *Academ* superfamily of DNA transposons from the fungus *Puccinia coronata*. Only one representative insertion is shown for each family. TSDs are colored in red. All termini and TSDs characterized in the study are shown in Supplementary Fig. S1

**Fig. 4:**
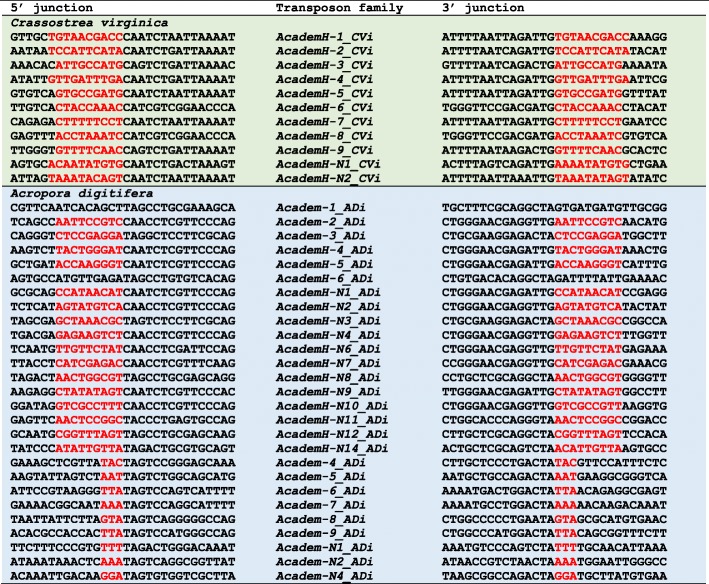
Termini and TSDs of *Academ* superfamily of DNA transposons from two animal species, *Crassostrea virginica* and *Acropora digitifera*. Only one representative insertion is shown for each family. TSDs are colored in red. No clear TSDs were observed for *Academ-1_ADi* and *AcademH-6_ADi*, while a short derivative of *Academ-1_ADi* is flanked by 9-bp TSDs (data not shown). All termini and TSDs characterized in the study are shown in Supplementary Fig. S2

It is reported that *AcademX* DNA transposons generate 3-bp or 4-bp TSDs [8, 16]. In contrast, *AcademH* generates relatively long TSDs. Fungal *AcademH* families generate 9-bp TSDs with some exceptions (Fig. 3, and Supplementary Fig. S1). Animal *AcademH* families generate 9 or 10-bp TSDs (Fig. 4 and Supplementary Fig. S2). In the genome of coral *A. digitifera*, both lineages of *Academ* DNA transposons (*AcademH* and *AcademX*) are present. *AcademH* is usually inserted with 9-bp TSDs. *AcademX* generates 3-bp TSDs the same as previously reported *AcademX* DNA transposons from animals.

Sequence comparison against reported non-autonomous TEs deposited in Repbase revealed that some of non-autonomous DNA transposons whose classification has not yet been determined are either *AcademX* or *AcademH* (Supplementary Table S3). DNA transposons with 8-bp or 9-bp TSDs show sequence similarity to *AcademH* termini while DNA transposons with 3-bp TSDs show sequence similarity to *AcademX* termini. One fungal species *Melampsora larici-populina*, closely related to *Puccinia*, and *Nematostella vectensis*, similarly to other cnidarian species, contain non-autonomous *AcademH* families (Table 1 and Supplementary Tables S2 and S3).

The presence of a pyrimidine (C or T) at the 5′ terminus and a purine (G or A) at the 3′ terminus is shared among almost all *Academ* families (Figs. 3 and 4). Some *Academ* families contain > 100-bp TIRs, represented by 526-bp TIRs of *AcademH-1_LoTr* and 575-bp TIRs of *AcademH-16_CVi*, while some have shorter than 10-bp TIRs; for example, *AcademH-2_PSt* and *AcademH-N13_PHor* have 8-bp TIRs.

### *AcademHP*, a sublineage of *AcademH* with PHD zinc fingers

Although no proteins from vertebrates were hit in the first iteration of PSI-BLAST search with the transposase of *AcademH-1_CVi* or *AcademH-6_PGr* as a query, the protein sequences from the four teleost fishes were hit in the second iteration. They are from the climbing perch *Anabas testudineus* (XP_026195931, XP_026196227, XP_026196228, XP_026196229), the California yellowtail *Seriola lalandi dorsalis* (XP_023286175, XP_023286176), the spiny chromis damselfish *Acanthochromis polyacanthus* (XP_022063315, XP_022063316, XP_022063317, XP_022063318)*,* and the rohu *Labeo rohita* (RXN19178, RXN19557). Besides these species, the genomes from a species of thornfishes *Cottoperca gobio*, the Siamese fighting fish *Betta splendens*, the bicolor damselfish *Stegastes partitus*, and the spotted seabass *Lateolabrax maculatus* contain related sequences (Supplementary Table S4). These proteins do not have all residues conserved among *AcademH* transposases (Fig. 2b, XP_026196227; and data not shown). Further investigation revealed that apparently intact *AcademH* transposons related to these proteins are present in the genomes of two deuterostomes: *AcademHP-1_SP* from the purple sea urchin *Strongylocentrotus purpuratus* and *AcademHP-1_SKow* from the acorn worm *Saccoglossus kowalevskii* (Fig. 2). These families encode 2 plant homeodomain (PHD) fingers between the second D and the last E catalytic residues (Figs. 1 and 2). One PHD finger contains 1 histidine residue sandwiched by 4 and 3 cysteine residues (Cys_4_-His-Cys_3_). PHD fingers share an ability to bind to tri-methylated lysines on histones [17], and thus, it is expected that the PHD fingers in the transposases of *AcademHP* families also bind to histones. Several copies of *AcademHP* families show 9-bp TSDs similarly to other *AcademH* families (Supplementary Fig. S3). One *AcademHP* sequence was also found as a single-copy sequence from the genome of *Priapulus caudatus*, although it encodes only one PHD finger (Fig. 2). Another protein encoded in the genome of *P. caudatus* (XP_014663285.1) contains 2 PHD fingers, although no TIRs flanked with recognizable TSDs were detected around the sequence encoding this protein. Thorough investigation revealed that other *AcademH* families from animals also contain a zinc finger motif between the second D and the last E catalytic residues, but they are CCHH-type (Fig. 2B2).

### *AcademH* and *AcademX*, two distant linages inside of *Academ* superfamily

HHpred analysis with *Academ* transposases did not indicate any specific relationships with other transposases. The transposase domains of *Academ* are considered to belong to the DDE transposases, and thus to the RNaseH fold, based on Yuan and Wessler [12] which reported the conserved motifs and residues among *Academ* transposases. With more divergent transposases included in this analysis, fewer conserved residues are recognized (Fig. 2b). Only 7 residues, including the proposed DDE triad, are conserved among diverse *Academ* transposases. Compared with other DDE transposases, the first catalytic D and the second catalytic D are very distant (138–192 residues apart) in *Academ* transposases. The conserved G/A/E/QxxH motif following the second catalytic D residue might correspond to C/DxxH motif in *MuDR*, *P*, *hAT*, *Kolobok* and *Dada*, predicted to be located at the beginning of insertion domain [12].

**Fig. 5:**
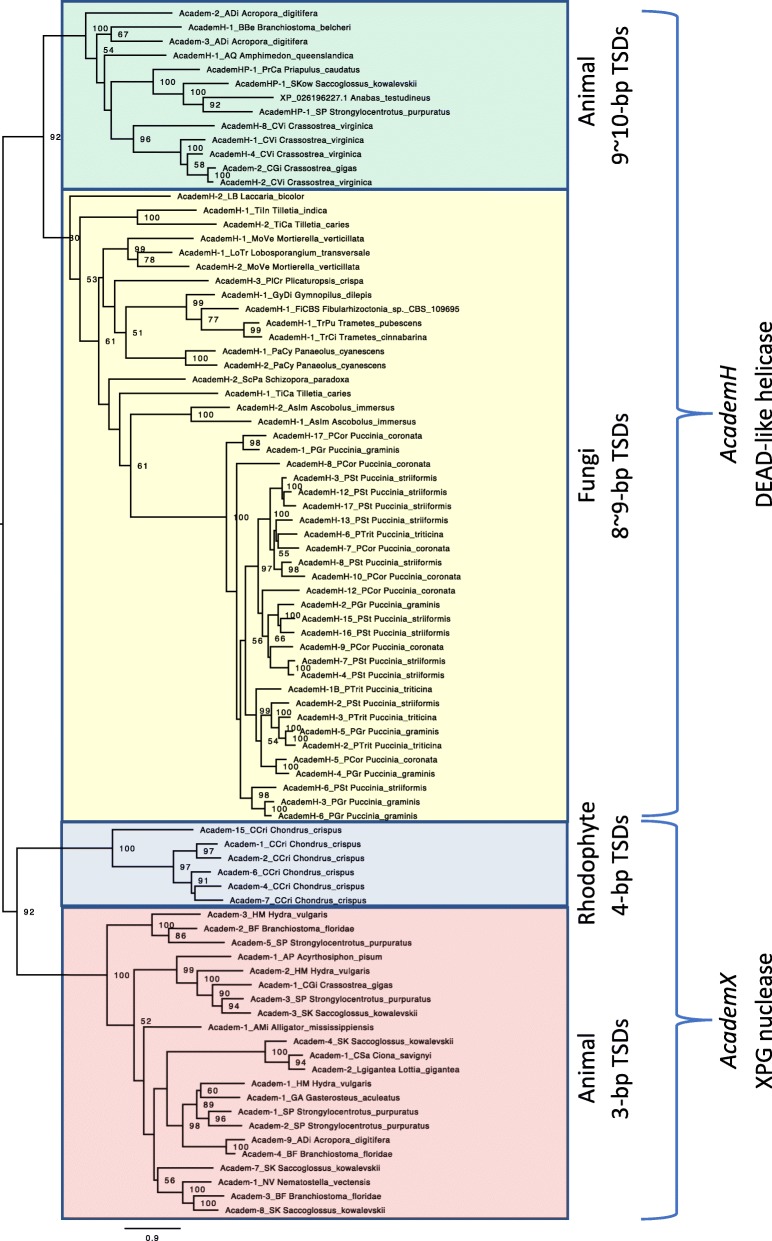
Phylogeny of *Academ* transposases. Four lineages recognized are highlighted in different colors. Bootstrap values of 100 replicates are shown at nodes when they are > 50. Host organisms of TEs are shown after TE names

The phylogenetic analysis revealed that *Academ* superfamily can be classified into two large groups, *AcademH* and *AcademX*, corresponding to the protein coding ability (Fig. 5). *AcademX* can be further divided into two lineages, consistent with the difference in TSD length and distribution. *AcademX* with 3-bp TSDs are distributed among animals. *AcademX* with 4-bp TSDs has been found only from the red alga *C. crispus*. Two clusters for *AcademH* correspond to the *AcademH* from fungi and animals. The three *AcademHP* families with the *AcademHP* transposase-like protein encoded on the genome of *A. testudineus* (XP_026196227.1) clustered together inside of animal *AcademH*. *AcademH* transposons from closely related organisms are often clustered together, for examples, three families from Mucoromycote fungi (*AcademH-1_LoTr*, *AcademH-1_MoVe* and *AcademH-2_MoVe*) or five families from the oysters in the genus *Crassostrea* (*AcademH-8_CVi*, *AcademH-1_CVi*, *AcademH-4_CVi*, *Academ-2_CGi*, and *AcdemH-2_CVi*). All *AcademH* families from the genus *Puccinia* are very closely related. However, deeper phylogeny of *AcademH* transposases is not consistent with their host phylogeny. Considering the small number of genomes from which *AcademH* families were characterized, and low bootstrap supports for deeper nodes, the contribution of horizontal transfer to the *AcademH* evolution remains to be investigated.

## Discussion

### The diversity and distribution of *Academ*

The *Academ* superfamily of DNA transposons has been found from three different groups of eukaryotes: animals, fungi and red algae. With a relatively small number of sequences, the phylogeny and structural characteristics of *Academ* are straightforward. The *AcademX* lineage encodes one large protein containing a transposase, an XPG nuclease and a putative zinc finger. It is distributed in animals and red algae. *AcademX* generates relatively short (3 or 4-bp) TSDs upon integration. The *AcademH* lineage encodes two proteins, one of which is a transposase and the other of which is a superfamily II helicase. *AcademH* generates relatively long (9 or 10-bp) TSDs upon integration. *AcademH* is distributed in animals and fungi. *AcademHP* is a sublineage inside of *AcademH* and this lineage encodes one or two PHD fingers between the second D and the last E catalytic residues. In vertebrates, the genomes of some teleost fishes keep remnants of *AcademHP* copies.

### Functional implications for helicase in the life cycle of *AcademH*

The length of TSDs is one of the hallmarks of superfamilies of DNA transposons. In general, inside of the superfamily of DNA transposons, the lengths of TSDs are not so divergent [4, 18]. Almost all of superfamilies show strict restriction of TSD lengths, which allows only 1-bp difference. As rare exceptions, the *hAT* superfamily shows TSDs of 5, 6 or 8-bp, and the *EnSpm* superfamily shows TSDs of 2-bp, 3-bp, or 4-bp. In contrast, inside of the *Academ* superfamily, *AcademX* generates 3-bp or 4-bp TSDs, while *AcademH* generates TSDs of 9 or 10 bps in length.

*AcademH* families encode a superfamily II helicase related to RecQ, while *AcademX* families encode an XPG nuclease. Mutually exclusive presence of helicase or nuclease in *Academ* transposons implies the functional similarity of these two enzymes in the life cycle of *Academ* transposons. RecQ helicase family works for various DNA repair pathways including homologous recombination and non-homologous end joining [19]. XPG nuclease families are needed to repair DNA damages by a process called nucleotide excision repair [20]. It can be speculated that helicase and nuclease encoded by *Academ* transposons are coupled with cellular proteins in DNA repair pathway during the transposition of *Academ* transposons. DDE transposases cleave DNA at both termini of DNA transposons [21]. The difference in how DNA repair pathway is recruited to resolve the transposition intermediate might dictate the junction structures different between *AcademX* and *AcademH*.

## Conclusions

The *Academ* superfamily of DNA transposons has 2 deep-branching lineages: *AcademX* and *AcademH*. Besides its transposase, *AcademH* encodes a superfamily II helicase, which may contribute to the generation of long TSDs.

## Methods

### Characterization of *Academ* DNA transposons

All genome sequences used in this study were downloaded from either of three websites: NCBI Assembly database (https://www.ncbi.nlm.nih.gov/assembly), UCSC Genome Browser (https://genome.ucsc.edu/), and OIST Marine Genomics Unit (http://marinegenomics.oist.jp/lingula/viewer/download?project_id=47), and listed in Supplementary Table S5.

Censor searches [13] using reported *Academ* sequences as queries against genomes were performed. Sequences showing similarity to *Academ* were clustered by BLASTCLUST in the NCBI Blast package. Censor searches were done with consensus sequence of each cluster and the hits with flanking sequences were extracted to characterize the complete repeat unit until TSDs were detected.

In parallel, RepeatModeler (http://www.repeatmasker.org/RepeatModeler/) and Repbase [1] were used for the initial screening of repetitive families with default parameters for all animal genomes used here except for *C. gigas*, *C. virginica*, *M. yessoensis, C. teleta*, *P. caudatus*, and *B. floridae*. Consensus sequences generated by RepeatModeler output with the annotation as *Academ* were chosen to reconstruct the second consensus sequences using the top 10 hits with the 1000-bp flanking sequences at both sides in the Censor search.

Single-copy sequences similar to *AcademH* families were annotated as *AcademH* transposons if > 10-bp TIRs and adjacent > 8-bp TSDs were detected within their 10,000-bp flanking sequences.

The consensus or single-copy representative sequences for all TE families reported here have been submitted to Repbase [1], and are also available in Supplementary Datasets S1 and S2.

### Protein structure and phylogenetic analyses

Protein coding regions were predicted from consensus sequences and representative single-copy sequences with Softberry FGENESH (http://www.softberry.com/berry.phtml?topic=fgenesh&group=programs&subgroup=gfind) [22], followed by manual curation with reference to predicted mRNA sequences available at NCBI website. NCBI CD-Search (https://www.ncbi.nlm.nih.gov/Structure/cdd/wrpsb.cgi) [23] was done to detect protein domains. HHpred (https://toolkit.tuebingen.mpg.de/tools/hhpred) [24] was used to find similar structures of respective proteins.

Multiple sequence alignment was done with MAFFT with linsi option [25]. *Academ* transposase domains were extracted following the definition in [12, 16]. Protein sequences with truncation or internal deletion inside of transposase domain were excluded from the analysis. The final dataset used for the phylogenetic analysis contains 86 sequences which are 319 to 541 residues in length (Supplementary Dataset S3). Maximum likelihood trees with bootstrap values of 100 replicates were constructed using PhyML [26] with the amino acid substitution model LG + G + I + F, which was chosen based on the best Akaike Information Criterion score. The phylogenetic trees were drawn with the aid of FigTree 1.3.1 (http://tree.bio.ed.ac.uk/software/figtree/).

## Supplementary information

**Additional file 1: Figure S1.** Termini and TSDs of newly characterized families of *Academ* from the fungus *Puccinia coronata*. **Figure S2.** Termini and TSDs of newly characterized families of *Academ* from two animal species, *Crassostrea virginica* and *Acropora digitifera*. **Figure S3.** Termini and TSDs of *AcademHP* families from animals. **Table S3.** Non-autonomous DNA transposons newly classified as *Academ*. **Table S4.***AcademHP* remnants found in teleost.

**Additional file 2 : Table S1.** Protein sequences showing similarity to *AcademH* transposases. **Table S2.** Characteristics of *AcademH* families. **Table S5.** Genome assembly sequences used in this study.

**Additional file 3 : Data S1.** Consensus sequences of multicopy *Academ* transposons characterized in this study. **Data S2.** Representative sequences of single-copy *Academ* transposons characterized in this study. **Data S3.** Protein multiple alignment of *Academ* transposase domains used for the phylogenetic analysis.

## Data Availability

All data generated or analyzed in this study are included in this published article and its supplementary information files. Consensus and single-copy representative sequences of TEs are also submitted to Repbase (http://www.girinst.org/repbase/).

## Abbreviations

TSD: Target site duplication
TIR: Terminal inverted repeat
PHD: Plant homeodomain
TE: Transposable element
LTR: Long terminal repeat
RAG1: Recombination activating gene 1
