# Supplementary material for: AcademH, a lineage of Academ DNA transposons encoding helicase found in animals and fungi
Source: Mob DNA. 2020 Apr 18;11:15. doi: 10.1186/s13100-020-00211-1 (PMC7165386; doi:10.1186/s13100-020-00211-1)
Supplement: Supplementary file 1 — Additional file 1: Figure S1. Termini and TSDs of newly characterized families of Academ from the fungus Puccinia coronata. Figure S2. Termini and TSDs of newly characterized families of Academ from two animal species, Crassostrea virginica and Acropora digitifera. Figure S3. Termini and TSDs of AcademHP families from animals. Table S3. Non-autonomous DNA transposons newly classified as Academ. Table S4.AcademHP remnants found in teleost. [file 13100_2020_211_MOESM1_ESM.pdf]

Figure S1. Termini and TSDs of newly characterized families of *Academ* from the fungus *Puccinia coronata*. TSDs are colored in red. Positions of TEs are shown in parentheses. Up to 20 sequences are shown for each family.

Puccinia coronata

AcademH-1_PCor
PGCI01000065.1_[144956-151367]
GGACGCACATTTTTCCGAACTTAACCCAAGCCCCTCGCCTGCCGCAGGCAGGGGCGCGCAGCGCCTCGCG//AGACCCTGCGGGGCTGCCCCCCGGGGGCTCTCACTCAGAGGCTTTGTTAAGCTCGACATTTTTCACGCTC
PGCI01000304.1_[2522-8929]
TTCTGATGAGTGAGGCGAACTTAACCCAAGCCCCTTGCCTGCCGCAGGCAGGGGCGCGCAGCGCCTCGCG//AGACCCTGCGGGGCTGCCCCCCGGGGGCTCTCACTCAGAGGCTTTGTTAAGCTCGTGAGTGAGGAGGGTT
PGCI01000703.1_[22681-29089]
AGTATGGAGACAACGCGAACTTAACCCAAGCCCCTCGCCTGCCGCAGGCAGGGGCGCGCAGCGCCTCGCG//AGACCCTGCGGGGCTGCCCCCCGGGGGCTCTCACTCAGAGGCTTTGTTAAGCTCGGAGACAACGGTGAGG
PGCI01000804.1_[40186-33807]
CGCCGCGCCTTTTGGCGAACTTAACCCAAGCCCCTCGCCTGCCGCAGGCAGGGGCGCGCAGCGCGACCTC//AGACCCTGCGGGGCTGCCCCCCGGGGGCTCTCACTCAGAGGCTTTGTTAAGCTCGGCCTTTTGGGAGTTG
PGCI01001031.1_[6359-12770]
GGACGCACATTTTTCCGAACTTAACCCAAGCCCCTCGCCTGCCGCAGGCAGGGGCGCGCAGCGCCTCGCG//AGACCCTGCGGGGCTGCCCCCCGGGGGCTCTCACTCAGAGGCTTTGTTAAGCTCGACATTTTTCACGCTC

AcademH-2_PCor
PGCI01000061.1_[42194-36049]
CAGGCCATTTGATCCCTAACTTAACGCAAGTCCATCGCTTTGGAGGGCCTGGGCCTGGGGTGGGCGCAAA//TTGCATCCGGGCCCTTCGGGCCCCCGATTACCATTGGAGGCTTAGTTAAGCTAGATTTGATCCTAGGGA
PGCI01000266.1_[73642-79789]
GGAGTGTATCCACCACTAACTTAACGCAAGTCCATCGCTTTGGAGGGCCTGGGCCTGGGCCTGGGGTGGG//TCGCATCCGGGCCCTTTGGGCCCCCAATTACCATTGGAGGCTTAGTTAAGCTAGATCCACCAAGGGCCA
PGCI01000656.1_[41733-35594]
CAGGCCATTTGATCCCTAACTTAACGCAAGTCCATCGCTTTGGAGGGCCTGGGCCTGGGGTGGGCGCAAA//TTGCATCCGGGCCCTTCGGGCCCCCAATTACCATTGGAGGCTTAGTTAAGCTAGATTTGATCCTAGGGA

AcademH-3_PCor
PGCI01000109.1_[231140-224926]
CCTTGGGACGTTTGTCTAGCTTAACACAAGCCCCTCCTTGTCGGAGGGGCCGGGTATTGCCAGGCCGGCG//CCGGTGCGCCGGGGTTCCCCCGGCCCTCCGAAGCGAGGGGATTGTGTTAAGCTAGGACGTTTGTGAGGAA
PGCI01000242.1_[44725-38513]
GGGACCGAGGTCTTCCTAGCTTAACACAAGCCCCTCCTTGTTGGAGGGGCCGGGTATTGCCAGGCCGGCG//CCGGCGCGCCGGGGTTCCCCCGGCCCTCCGAAGCGAGGGGATTGTGTTAAGCTAGGAGGTCTTCCCATTT
PGCI01000258.1_[101603-107817]
CCTTGTGGTGCTACTCTAGCTTAACACAAGCCCCTCCTTGTCGGAGGGGCCGGGTATTGCCAGGCCGGCG//CCGGCGCGCCGGGGTTCCCCCGGCCCTCCGAAGCGAGGGGATTGTGTTAAGCTAGGGTGCTACTGCGGCG
PGCI01000681.1_[32706-26492]
GGGACTGAGGTCTTCCTAGCTTAACACAAGCCCCTCCTTGTTGGAGGGGCCGGGTATTGCCAGGCCGGCG//CCGGCGCGCCGGGGTTCCCCCGGCCCTCCGAAGCGAGGGGATTGTGTTAAGCTAGGAGGTCTTCCCATTT
PGCI01000710.1_[91354-97548]
CCTTGTGGTGCTACTCTAGCTTAACACAAGCCCCTCCTTGTCGGAGGGGCCGGGTATTGCCAGGCCGGCG//CTCGCTTCGCGAGGCGCCCCCGGCCCTCCGAAGCGAGGGGATTGTGTTAAGCTAGGGTGCTACTGCGGCG

AcademH-4_PCor
PGCI01000041.1_[362047-368041]
GACCATGTACAGTGGCTAGCTTAACCCAAGCCCCTCGCTTCAGAGGGCCAGGTATTGCCTGGCGGCGCGA//GAGCCCTCCGGGCGGGTCCCCCGGACCCTCCGTTCGAGAGGCTTAGTTAGGCTAGGTACAGTGGCTGGAA
PGCI01000077.1_[300954-294960]
ACCCCAGAGACCCCCCTAGCTTAACCCAAGCCCCTCGCTTCAGAGGGCCAGGTATTGCCTGGCGGCGCGA//GAGCCCTCCGGGCGGGTCCCCCGGACCCTCCGTTCGAGAGGCTTAGTTAGGCTAGGAGACCCCCTATATA
PGCI01000992.1_[46282-40286]
TCCCGCACTTTTGGACTAGCTTAACCCAAGCCCCTCGCTTCAGAGGGCCAGGTATTGCCTGGCGGCGCGA//GAGCCCTCCGGGCGGGTCCCCCGGACCCTCCGTTCGAGAGGCTTAGTTAGGCTAGACTTTTGGATCCCGC
PGCI01001013.1_[36272-42266]
GACCATGTACAGTGGCTAGCTTAACCCAAGCCCCTCGCTTCAGAGGGCCAGGTATTGCCTGGCGGCGCGA//GAGCCCTCCGGGCGGGTCCCCCGGACCCTCCGTTCGAGAGGCTTAGTTAGGCTAGGTACAGTGGCTGGAA

AcademH-5_PCor
PGCI01000059.1_[152087-158486]
GAAAAGGCTTGAACGCTAGCTTAACTAAGCCTCGCGAGCACGAGCACGAAGTGCGAGTGCGAGCAAAGGC//AGCCTCGCTCGCACTCGCACTTCGTGCTCGTGCTCGCAAGGCTTAGTTAAGCTAGGCTTGAACGGCCGTA
PGCI01000076.1_[50568-44173]
CATACCACTTGAATACTAGCTTAACTAAGCCTCGCGAGCACGAGCACGAAGTGCGAGTGCGAGCGAGGCT//AGCCTCGCTCGCACTCGCACTTCGTGCTCGTGCTCGCGAGGCTTAGTTAAGCTAGACTTGAATAGAAGTG
PGCI01000889.1_[51199-44803]
GGCAACAGGTTCATTCTAGCTTAACTAAGCCTTGCGAGCACGAGCACGAAGTGCGAGTGCGAGCGAGGCT//AGCCTCGCTCGCACTCGCACTTTGTGCTCGTGCTCGCGAGGCTTAGTTAAGCTAGAGGTTCATTGTTGAA

AcademH-6_PCor
PGCI01000146.1_[198602-191669]
GGCTGAGGTCAGTTTCTAACTTAACTAAGTCCCACCCTCCGTCCGCGGCCGAGTGGTTTAGCATGTGCTT//GTCCCTGCGGGACAAGTCCCCCCTCCGACATAACAGAGGGACTTAGTTAAGTTAGGGTCAGTTTCGTCCT
PGCI01000447.1_[28647-21684]
AACGGTGTATAAACCCTAACTTAACTAAGTCCCACCCTCCGTCCGCGGCCGAGTGGTTTAGCCTGTGCTT//GTCCCTGCGGGACAAGTCCCCCCTCCGACATAACAGAGGGACTTAGTTAAGTTAGGTATAAACCACTCTC
PGCI01000652.1_[33638-26674]
AGGTCCACTGCAACACTAACTTAACTAAGTCCCACCCTCCGTCCGCGGCCGAGTGGTTTAGCATGTGCTT//GTCCCTGCGGGACAAGTCCCCCCTCCGACATAACAGAGGGACTTAGTTAAGTTAGACTGCAACAGTGGGT
PGCI01000819.1_[3472-10437]
ATGTACATACATTCCCTAACTTAACTAAGTCCCACCCTCCGTCCGCGGCCGAGTGGTTTAGCATGTGCTT//GTCCCTGCGGGACAAGTCCCCCCTCCGACATAACAGAGGGACTTAGTTAAGTTAGATACATTCCCCACTT

AcademH-7_PCor
PGCI01000057.1_[11836-5549]
GGGATAGGCTCAACGCTAACTTAACAACAAGTCCCTCGAATCGGAGGGCCTGGGCGGGCGCAAAGCGCCC//TTCGCATCCGGGCCCTTTGGGCCCCCGATCCCATCGGAGGCTTAGTTAAGCTAGGGCTCAACGTATATG
PGCI01000233.1_[142080-135790]
GGGATAGGCTCAACGCTAACTTAACAACAAGTCCCTCGAATCGGAGGGCCTGGGCGGGCGCAAAGCGCCC//TTCGCATCCGGGCCCTTTGGGCCCCCGATCCCATCGGAGGCTTAGTTAAGCTAGGGCTCAACGTATATG
PGCI01000617.1_[100323-106629]
AAAAGCAATTACAACCTAACTTAACAACAAGTCCCTCAAATTGGAGGGCCTGGGCGGGCGCGAAGCGCCC//TTCGCATCCGGGCCCTTCGGGCCCCCAATCCCATTGGGGGGCTTAGTGAAGCTAGAATTACAACCCGGAC

AcademH-8_PCor
PGCI01000236.1_[116731-109987]
TTAAGGCACTTACGGCGAACTTAACAAAGTCCCGCTGGGAGGGCCCCCGGCCCGCGCCAGCGGGACTTAT//TCCCCGGTGGGGTTTTTCACCCCCGGGCAACGAGCCAAGGACTTAGTTAAGTTTGCACTTACGGTAGAAA
PGCI01000779.1_[46486-39754]
TTAAGGCACTTACGGCGAACTTAACAAAGTCCCGCTGGGAGGGCCCCCGGCCCGCGCCAGCGGGACTTAT//TCCCCGGTGGGGTTTTTCACCCCCGGGCAACGAGCCAAGGACTTAGTTAAGTTTGCACTTACGGTAGAAA

AcademH-9_PCor
PGCI01000237.1_[78845-82788]
CACCACCACCCGCACGAACTTAACAACAAGCCCCTATTCCCTGCTGTGCGCGGCTTATGCATGTGGGTGA//CCTCTCTGTGAGACAGAGACCCTGCGGGGCTCCTCCAGGAGGCTTTGTTAAGCTCGGCAAGTCTTGCGCC
PGCI01001038.1_[15743-9062]
ATATGTGCGGCATTCGAACTTAACAGCAAGCCCCTATTCCCTGCTGTGCGCGGCTTATGCATGTGGGTGA//CCTCTCTGTGAGACAGAGACCCTGCGGGGCTCCTCCAGGAGGCTTTGTTAAGCTCGTGCGGCATTGTGCT

AcademH-10_PCor
PGCI01000025.1_[92364-98915]
GTGGAGCCCAAGGAGCTAGCTTAACACAAGCACCTCCTTGTCAGAGGCGCCTGGTATCGCCAGGCGGGGG//CTCGCTTCGCGAGGCGCCCCCGGCCCTCCGAAGCGAGGGGATTGTGTTAAGCTAGAAGGAGAGATGAACT
PGCI01001069.1_[20777-27328]
GTGGAGCCCAAGGAGCTAGCTTAACACAAGCACCTCCTTGTCAGAGGCGCCTGGTATCGCCAGGCGGGGG//CTCGCTTCGCGAGGCGCCCCCGGCCCTCCGAAGCGAGGGGATTGTGTTAAGCTAGAAGGAGAGATGAACT

AcademH-11_PCor
PGCI01000089.1_[169178-162307]
GAATGGATTTCTTCACAAACTTATCAAAGTCCCAGACCCTGCCTGGTCGCCACGTTTAGTGTGGTGCTGC//AGCCTCGCTTGCACCTCACGCCTCTCGGCGGTGCTCGCGGGCTTTGTTACGCTCGATTTCTTCAATCATC
PGCI01000759.1_[37404-30533]
GAATGGATTTCTTCACAAACTTATCAAAGTCCCAGACCCTGCCTGGTCGCCACGTTTAGTGTGGTGCTGC//AGCCTCGCTTGCACCTCACGCCTCTCGGCGGTGCTCGCGGGCTTTGTTACGCTCGATTTCTTCAATCATC

AcademH-12_PCor
PGCI01000197.1_[138671-132496]
ATCGTACGTTGAGCCGAACTTAACACAAGTCCCTCGGCCTGCAGCAGGCAGGCGCCTCCCGTCAGGGAGG//TTTGCGACGCGGGGTCCCCCCGGGACCCTCCAATCGAGAGGCTTAGTTAAGCTTGCGTTGAGCCTACAGT
PGCI01000702.1_[82403-76228]
GCAGTGTGTACAGTCGAACTTAACACAAGTCCCTTGGCCTGCCGCAGGCAGGCGCCTCCCGTCAGGGAGG//TTTGCGACGCGGGGTCCCCCCGGGACCCTCCAATCGAGAGGCTTAGTTAAGCTTGCGTTGAGCCTACAGT

AcademH-13_PCor
PGCI01000002.1_[630013-636400]
AAATGGGTGTAAATGCTAACTTAACACAAGTCCCGCCCGACCGCGGGAGGGACTTGCAATGTATGTCACA//GAGCGCTCCGCGCGGGGTCCCCCGGACCCTCCGTTCGAGTGGCTTTGTTAAGCTTGTGTAAATGGAGGAC
PGCI01000009.1_[754354-766839]
ATATGCAACCGCGGTGTAACTTAACACAAGTCCCTCCCGACCGCGGGAGGGCGGCGAAGCCGCCCTCCTG//GAGCGCTTCGCGCGGGGTCCCCCGGACCCTCCGTTCGAGTGGCTTTGTTAAGCTCGACCTATCTGGACAG
PGCI01000161.1_[28490-20378]
AAGGATACAGGGCTGCTAACTTAACACAAGTCCCGCCCGACCGCGCGGGAGGGACTTTCAATGTATGTCA//GAGCGCTCCGCGCGGGGTCCCCCGGACCCTCCGTTTGAGTGGCTTTGTTAAGCTCGACAGGGCTGTACAG
PGCI01000672.1_[32690-39075]

AAATGGGTGTAAATGCTAACTTAACACAAGTCCCGCCCGACCGCGGGAGGGACTTGCAATGTATGTCACA//GAGCGCTCCGCGCGGGGTCCCCCGGACCCTCCGTTCGAGTGGCTTTGTTAAGCTTGTGTAAATGGAGGAC

AcademH-14_PCor
PGCI01000316.1_[64813-58721]
TAGCCCTTTAAGCTGCTAACTTAACACAAGTCCTGGGGGCTGCGGCGCTGTGTAAGCACTGTTGCTTGCG//GCGCTCCGCGCGGGGTCCCCGGGACCCTCCGTTCGCGAGGCTTATGTTAAGCTAGTTTAAGCTGAGTCTG
PGCI01001193.1_[13458-7363]
TAGCCCTTTAAGCTGCTAACTTAACACAAGTCCTGGGGGCTGCGGCGCTGTGTAAGCACTGTTGCTTGCG//GCGCTCCGCGCGGGGTCCCCGGGACCCTCCGTTCGCGAGGCTTATGTTAAGCTAGTTTAAGCTGAGTCTG

AcademH-15_PCor
PGCI01000058.1_[40551-33361]
GAGCTGGGAACTAATCAAACTTAACACAAGTCCCTTGCCTGCCGGAGGCAGGGGCGCAAAGCGCGACCTC//CTCTGTGAGACAGATACCCTGCGGGGCCCTCACTTGAGAGGCTTTGTTAAGCTCGGGAACTAATTGGGTT
PGCI01000614.1_[28142-20953]
GAGCTGGGAACTAATCAAACTTAACACAAGTCCCTTGCCTGCCGGAGGCAGGGGCGCAAAGCGCGACCTC//CTCTGTGAGACAGATACCCTGCGGGGCCCTCACTTGAGAGGCTTTGTTAAGCTCGGGAACTAATTGGGTT

AcademH-16_PCor
PGCI01000213.1_[141445-148248]
AAATTGATGCCTGTGCAAACTTAACAACAAGTCTGCCTGCTTGCAGGGCCGGGCGGGGCTTAGACGCGTT//TGGACAGCTTCGCTGCTTGCCTCCACTCCTCGAAGTTGAGGCTTAGTTAAGGTTGATGCCTGTGAGTCAA
PGCI01000923.1_[38223-45024]
AAATTGATGCCTGTGCAAACTTAACAACAAGTCTGCCTGCTTGCAGGGCCGGGCGGGGCCTAGACGCGTT//TGGACAGCTTCGCTGCTTGCCTCCACTCCTCGAAGTTGAGGCTTAGTTAAGGTTGATGCCTGTGAGTCAA

AcademH-17_PCor
PGCI01000153.1_[111495-118261]
AGGCACATGGGGGGCCTAACTTAACAAAGTCCCTCTGTAACTTTGTGCGAGTCCCAAAGGGCCTTTGAAG//CTTCGGAGGCCCTTCGGGACTCGCACGAAGTTACAGAGGGACTTTGTTAAGTTAGATGGGGGGCCATTTT

AcademH-N1_PCor
PGCI01000021.1_[303006-302131]
TTGTAAGTGGCATCTCTAACATAAGTAAGTCCCCTCTGGGACGGGCCCCGCAGGGCCCCTCGTGAGAGGG//GGCGCTCCGTGGGGTTCCCGGCGCCTGCCACCAGGGTGGACTTGTGTTAAGTTAGGTGGCATCTCAAAGT
PGCI01000035.1_[363059-363873]
AGGGTGAGCATCCATCTAACTTAACTAAGTCCCCTCTGGGACGGGCCCCGCAGGGCCCCTTGTGAGAGGG//GGCGCTCTGCGGGGTTCCCGGCGCCTGCCACCAGGGTGGACTTGTGTTAAGTTAGAGCATCCATGCTGAT
PGCI01000050.1_[304837-305651]
GAAGCAAGCAGATACCTAACTTAACTAAGTCCCCTCTGGGATGGGCCCTGCAGGGCCCCTTGTGAGAGGG//GGCGCTCCGCGGGGTTCCCGGCGCCTGCCACCAGGGTGGACTTGTGTTAAGTTAGAGCAGATACATACTC
PGCI01000094.1_[136872-137689]
GGGGTGCATACTCTACTAACTTAACTAATTAGTCCCCTCTGGGAAGAGCCCCGCAGGGCCCCTCGTGAGA//GGCGCTCCGCGGGGTTCCCGGCGCCTGCCACCAGGGTGGACTTGTGTTAAGTTAGCATGCTCTAGGGAAA
PGCI01000122.1_[30311-31124]
ACGTCATAAACAAGTCTAACTTAACTAAGTCCCCTCTGAGACGGGCCCCGCAGGGCCCCTCGTGAGAGGG//GGCGCTCCGCGGGGTTCCTGGCGCCTGCCACCAGGGTGGACTTGTGTTAATTTAGTAAACAAGTGGTACT
PGCI01000185.1_[48388-49203]
TGACTGCTGTTGAGTCTAACTTAACTAAGTCCCCTCTGGGAAGGGCCCCGCAGGGCCCCTCGTGAGAGGG//GGCGCTCCGCGGGGTTCCCGGCGCCTGCCACCAGGGTGGACTTGTGTTAAGTTAGCTGTTGAGTGAGGTC
PGCI01000237.1_[17740-16927]
AAGTAGGGGTGTGTGCTAACTTAACTAAGTCCCCTCTGGGACGGGCCCCGCTGGGCCCCTTGTGAGAGGG//GGCGCTCCGCGGGGTTCCCGGCGCCTGCCACCAGGGTGAACTTGTGTTAAGTTAGGGGTGTGTGGAGGAG
PGCI01000290.1_[30319-29506]
TGGAGGTGTCAACACCTAACTTAACTAAGTCCCCTCTGGGACGGGCCCCGCAGGGCCCCTCGGGAGAGGG//GGCACTCCGCGGGGTTCCCTGCACCTGCCACCAGGGTGGACTTGTGTTAAGTTAGTGTCAACACCTTGAT
PGCI01000425.1_[22294-23095]
ACACGTGAAAAGGACCTAACTTAACTAAGTCCCCTCTGGGACGGGCCCCTGATGAGAGGGGACTTATGAC//GGCGCTCTGCGGGGTTCCCAGCGCCTGCCACCAGGGTGGACTTGTGTTAAGTTAGGAAAAGGACCAGCAT
PGCI01000521.1_[15283-16100]
GTGTTGTGGTAGTATCTAACTTAACTAAGTCCCCTCTGGGACGGGCCCCGCAGGGCCCCTCGTGAGAGGG//GGCGCTCTGCGGGGTTCCCGGCGCCTGCCACCAGGGTGGACTTGTGTTAAGTTGGTGGTAGTATCATGCC
PGCI01000607.1_[129700-128825]
TTGTAAGTGGCATCTCTAACATAAGTAAGTCCCCTCTGGGACGGGCCCCGCAGGGCCCCTCGTGAGAGGG//GGCGCTCCGTGGGGTTCCCGGCGCCTGCCACCAGGGTGGACTTGTGTTAAGTTAGGTGGCATCTCAAAGT
PGCI01000620.1_[63524-64341]
GGGGTGCATACTCTACTAACTTAACTAATTAGTCCCCTCTGGGAAGAGCCCCGCAGGGCCCCTCGTGAGA//GGCGCTCCGCGGGGTTCCCGGCGCCTGCCACCAGGGTGGACTTGTGTTAAGTTAGCATGCTCTAGGGAAA
PGCI01000658.1_[103380-104194]
GAAGCAAGCAGATACCTAACTTAACTAAGTCCCCTCTGGGATGGGCCCTGCAGGGCCCCTTGTGAGAGGG//GGCGCTCCGCGGGGTTCCCGGCGCCTGCCACCAGGGTGGACTTGTGTTAAGTTAGAGCAGATACATACTC
PGCI01000669.1_[71661-72475]
AGGGTGAGCATCCATCTAACTTAACTAAGTCCCCTCTGGGACGGGCCCCGCAGGGCCCCTTGTGAGAGGG//GGCGCTCTGCGGGGTTCCCGGCGCCTGCCACCAGGGTGGACTTGTGTTAAGTTAGAGCATCCATGCTGAT
PGCI01000784.1_[14226-13413]
AAGTAGGGGTGTGTGCTAACTTAACTAAGTCCCCTCTGGGACGGGCCCCGCTGGGCCCCTTGTGAGAGGG//GGCGCTCCGCGGGGTTCCCGGCGCCTGCCACCAGGGTGAACTTGTGTTAAGTTAGGGGTGTGTGGAGGAG
PGCI01000858.1_[19358-20159]
ACACGTGAAAAGGACCTAACTTAACTAAGTCCCCTCTGGGACGGGCCCCTCATGAGAGGGGACTTATGAC//GGCGCTCTGCGGGGTTCCCAGCGCCTGCCACCAGGGTGGACTTGTGTTAAGTTAGGAAAAGGACCAGCAT
PGCI01001027.1_[30792-31607]
TGACTGCTGTTGAGTCTAACTTAACTAAGTCCCCTCTGGGAAGGGCCCCGCAGGGCCCCTCGTGAGAGGG//GGCGCTCCGCGGGGTTCCCGGCGCCTGCCACCAGGGTGGACTTGTGTTAAGTTAGCTGTTGAGTGAGGTC
PGCI01001028.1_[26106-26919]
ACGTCATAAACAAGTCTAACTTAACTAAGTCCCCTCTGAGACGGGCCCCGCAGGGCCCCTCGTGAGAGGG//GGCGCTCCGCGGGGTTCCTGGCGCCTGCCACCAGGGTGGACTTGTGTTAATTTAGTAAACAAGTGGTACT
PGCI01001141.1_[15327-16144]
GTGTTGTGGTAGTATCTAACTTAACTAAGTCCCCTCTGGGACGGGCCCCGCAGGGCCCCTCGTGAGAGGG//GGCGCTCTGCGGGGTTCCCGGCGCCTGCCACCAGGGTGGACTTGTGTTAAGTTGGTGGTAGTATCATGCC
PGCI01001428.1_[5762-6561]
AAGAGGGCTCTATATCTAACTTAACTAAGTCCCCTCTGGGACGGGCCCTGCAGGGCCCCTCGTGAGAGGG//GGCACTCCGCAGGGTTCCCGGCGCCTGCCACCAGGGTGGACTTGTGTTAAGTTAGGCTCTATATATACTA

AcademH-N2_PCor
PGCI01000029.1_[327719-328510]
TGGACGAGTGCGCAGCTAGCTTAACCCAATCCCCTTGCTTCAGAGGGCCAGGTATTGCCTGGCGGCGCGA//GAGCCCTCCGGGAGGGTCCCCCGGACCCTCCGTTTGAGAGGCTTAGTTAGGCTAGAGTGCGCAGGTGGCA
PGCI01000033.1_[48246-47454]
AGCCAACGTTGCCCCCTAGCTTAACCCAATCCCCTTGCTTCAGAGGGCCAGGTATTGCCTGGCGGCGCAA//GAGCCCTCCGGGCGGGTCCCCCGGACCCTCCGTTCGAGAGGCTTAGTTAGGCTAGCGTTGCCCCCACATG
PGCI01000046.1_[126632-127423]
CTTCCCTTTTACCTGCTAGCTTAACCCAATCCCCTTGCTTCAGAGGGCCAGGTATTACCTGGCGGCGCGA//GAGCCCTCCGGGCGGGTCCCCCGGACCCTCTGTTCGAGAGGCTTAGTTAGGCTAGTTTTACCTGTAGGAT
PGCI01000050.1_[222118-222909]
AAGGAGAAATCATCCCTAGCTTAACCCAATCCCCTTGCTTCAGAGGGCCAGGTATTGCCTGGCGGCGCGA//GAGCCCTCCGGGCGGGTCCCCTGGACCCTCCGTTCGAGAGGCATAGTTAGGCTAGAAATCATCCTCGGTG
PGCI01000059.1_[269133-269924]
TTGTTAACCATCAAGCTAGCTTTACCCCAATCCCCTTGCTTCAGAGGGCCAGGTATTGCCTGGCGGCGCG//AGAGCCCTCCGGGCGGGTCCCCGGACCCTCCGTTCGAGAGGCTTAGTTAGGCTAGACCATCAAGCAGGGA
PGCI01000073.1_[176862-176088]
AACTTGGGTGTGCAACTAGCTTAACCCAATCCCCTTGCTTCAGAGGGCCAGGTATTGCCTGGCGGCGCAA//GAGCCCTCCGGGCGGGTCCCTTGGACCCTCCGTTCAAGAGGCTTAGTTAGGCTAGGGTGTGCAACACCGA
PGCI01000076.1_[33876-34667]
CCGTTGGGTACAGTTCTAGCTTAACCCAATCCCCTTGCTTCAGAGGGCCAGGTATTGCCTGGCGGCGCGA//GAGCCCTCCGGGCGGGTCCCCCGGACCCTCCGTTCGAGAGGCTTAGTTAGGCTAGGGTACAGTTGCGGAC
PGCI01000126.1_[80136-79345]
GATTGGGGTGTTGATCTAGCTTAACCCAATCCCCTTGCTTCAGAGGGCCAGGTATTGCCTGGCGGCGCGA//GAGCCCTCCGGGCGGGTCCCCCGGACCCTCCGTTCAAGAGGCTTAGTTAGGCTAGGGTGTTGATGTAGTG
PGCI01000131.1_[66969-67760]
CCAGCAACTCCCACACTAGCTTAACCCAATCCCCTTGCTTCAGAGGGCCAGGTATTGCCTGGCGGCGCGA//GAGCCCTCCGGGCGGGTCCCCCGGACCCTCCGTTCGAGAGGCTTGGTTAGGCTAGCTCCCACAGGGGTCG
PGCI01000148.1_[110233-109442]
AGGCACTGCTAGTGGCTAGCTTAACCCAATCCCCTTGCTTCAGAGGGCCAGGTATTGCCTGGCGGCACGA//GAGCCCTCCGGGCGGGTCCCCCGAACCCTCCGTTCGAGAGGCTTAGTTAGGCTAGTGCTAGTGGCTGGGA
PGCI01000235.1_[19272-18482]
TGCCCGGTCTCAGTGCTAGCTTAACCCAATCCCCTTTCTTCAGAGGGCCAGGTATTGCCTGGCGGCGCAA//GAGCCCTCCGGGCGGGTCCCCCGGACCCTCTGTTTGAGAGGCTTAGTTAGGCTAGGTCTCAGTGTTGGTA
PGCI01000252.1_[41740-42517]
CGGCTTCCTGCGGAACTAGCTTAACCCAATCACCTTGCTTCAGAGGGCCAGGTATTGCCTGGCGGCGCGA//GAGCCCTCCGGGAGAGTCCCCCGGACCCTCCGCTCAAGAGGCTTAGTTAGGCTAGCCTGTGGAAGCGCCT
PGCI01000261.1_[96046-96835]
TCGCAGGCTGCAGAGCTAGCTTAACCCAATCCCCTTGCTTCAGAGGGCCAGGTATTGCCTGGCGGCGCGA//GAGCCCTCCGGGAGGGTCCCCCGGACCCTCCGTTTGAGAGGCTTAGTTAGGCTAGGCTGCAGAGTGGCTG
PGCI01000277.1_[73189-72398]
TCGGCACCTGTTGCCCTAGCTTAACCCAATCCCCTTGCTTCAGAGGGCCAGGTATTGCCTGGCGGCGTGA//GAGCCCTCCGGGCGGGTCCCCCGGACCCTCCGTTTGAGAGGCTTAGTTAGGCTAGCCTGTTGCCTAGGGA
PGCI01000340.1_[77488-78279]
AGCGCCAACTGCAGCCTAGCTTAACCCAATCCCCTTGCTTCAGAGGGCCAGGTATTGCCTGGCGGCGCGA//GAGCCCTCCGGGCGGGTCCCCCGGACCCTCCGTTCGAGAGGCTTAGTTAGGCTAGCTGCAGCCAGCCCTG
PGCI01000408.1_[2412-1621]
GTCCGTAGAGAGAGCCTAGCTTAACCCAATCCCCTTGCTCCAGAGGGCCAGGTATTGCCTGGCGGCGCGA//GAGCCCTCCGGGCGGGTCCCCCGGACCCTCCGTTTGAGAGGCTTAGTTAGGCTAGAGAGAGAGCGGTCCA
PGCI01000415.1_[44845-44054]
AGATGAGGAAAGGGGCTAGCTTAACCCAATCCCCTTGCTTCAGAGGGCCAGGTATTGCCTGGCGGCGCGA//GAGCCCTCCGGGCGGGTCCCCCGGACCCTCCGTTCGAGAGGCTTAGTTAGGCTAGGGAAAGGGGCAGGGA
PGCI01000446.1_[26088-26879]
AGATGAGGAAAGGGGCTAGCTTAACCCAATCCCCTTGCTTCAGAGGGCCAGGTATTGCCTGGCGGCGCAA//GAGCCCTCCGGGCGGGTCCCCCGGACCCTCCGTTCGAGAGGCTTAGTTAGGCTAGGGAAAGGGGCAGGGA
PGCI01000610.1_[92321-91547]
AACTTGGGTGTGCAACTAGCTTAACCCAATCCCCTTGCTTCAGAGGTCCAGGTATTGCCTGGCGGCGCAA//GAGCCCTCTGGGCGGGTCCCCCGGACCCTCCGTTCAAGAGGCTTAGTTAGGCTAGGGTGTGCAACACCGA
PGCI01000677.1_[74422-73631]
GATTGGGGTGTTGATCTAGCTTAACCCAATCCCCTTGCTTCAGAGGGCCAGGTATTGCCTGGCGGCGCGA//GAGCCCTCCGGGCGGGTCCCCCGGACCCTCCGTTCGAGAGGCTTAGTTAGGCTAGGGTGTTGATGTAGTG

AcademH-N3_PCor
PGCI01000010.1_[658468-659179]
ACGGGTAGGGTGATGCTAACTTAACACAAGTCCCTCCAATCGGAGGGCCTGGTAGCCTGGCTCGCGGCAA//GAGCCCTCCGGGCGGGTCCCCCAGACCCTCCGTTCAAGAGGCTTTGTTAAGCTAGAGGGTGATGCATCTT
PGCI01000011.1_[235103-234352]
ATGGTGGGCCAGCAGCTAACTTAACACAAGTCCCTCCAATTGGAGGGCCTGGTAACCTGGGCGGGCGCAA//GAGCCCTCCGGGCGGGTCCCCCGGACCCTCCGTTCGAGAGGCTTTGTTAAGCTAGGGCCAGCAGCGTGTA
PGCI01000011.1_[236877-236118]
ATCCCACCATAGGACCTAACTTAACACAAGTCCCTCCAATCGGAGGGCCTGGTAACCTGGGCAGGTGCAA//GAGCCCTCCGGGCAGGTCCCCCGGACCCTCCGTTTGAGAGGCTTAGTTAGGCTAGCCATAGGACCAGGAA
PGCI01000024.1_[35771-36528]
CACTCACTTTGAACACTAACTTAACACAAGTCCCTCCAATCGGAGGGCCTGGCAACCTGGGCGGGCGCGA//GAGCCCTCCGGGCGGGTCCCCCGGACCCTCCGTTTGAGAGGCTTTGTTGAGCTAGCTTTGAACACAGATT
PGCI01000067.1_[122865-123605]
CAGCACGGAGTAGCCCTAACTTAACACAAGTCCCTCCAATCAGAGGGCCTGGTAACCTGGGCGGGCACAA//GAGCCCTCCGGGCGGGTCCCCCGGACCCTCCGTTCGAGAGGCTTAGTTAGGCTAGGGAGTAGCCTGAGCG

PGCI01000134.1_[171100-171857]
CAGTAGCCAACACTTCTAACTTAACACAAGTCCCTCCAATCGGAGGGCCTGGTAACCTGGGCGGGCGCGA//GAGCCCTCCGGGCGGGTCCCCCGGACCCTCCGTTCGAGAGGCTTAGTTAGGCTAGCCAACACTTGGGGTT
PGCI01000201.1_[141089-141846]
CGGGCGCGCGCCACCCTAACTTAACACAAGTCCCTCCAATCGGAGGGCCTGGTAACCTGGGCGGGCGCAA//GAGCCCTCCGGGCGGGTCCCCCGGACCCTCCGTTCAAGAGGCTTTGTAAAGCTAGCGCGCCACCGGCCGC
PGCI01000234.1_[35308-36047]
ACCCCCCTAATAGACCTAACTTAACACAAGTCCCTCCAATCAGAGGTCCTGGTAACCTGGGCGGGCGCAA//GAGCCCTCCGGGCGGGTCCCCCGGACCCTCCATTCGAGAGGCTTTGTTAAGCTAGCTAATAGACATGCAT
PGCI01000261.1_[47928-47189]
TGAAGGAGTAATCCCCTAAATTAACACAAGTCCCTCCAATTGGAGGGCCTGGTAACCCCCTCGCCGCAAA//GAGCCCTCCGGGCGGGTCCCCCGGACCCTCCATTTGATAGGCTTAGTTAGGCTAGAGTAATCCCCGGCGC
PGCI01000264.1_[74585-75341]
AACACCTCTCTGGCTCTAACTTAACACAAGTCCCTCCAATCGGAGGGCCTGGTAACCTGGGTGGGCGCAA//GAGACCTCCGGGCGGGTCCCCCGGACCCTCCGTTCGAGAGGCTTTGTTAAGCTAGTCTCTGGCTCATCAT
PGCI01000400.1_[4509-3752]
AACATGGCTGGTCAACTAACTTAACACAAGTCCCTCCAATCGGAGGGCCTGGTAACCTGGGCGGGCGCAA//GAGCCCTCCGGGCGGGTCCCCCGGACCCTCCATTCGAGAGGCTTAGTTAGGCTAGGCTGGTCAAGCAGTT
PGCI01000610.1_[142435-141678]
GCCCGCTTATCACCCCTAACTTAACACAAGTCCCTCCAATCGGAGGGCCTGGTAGCCTGGGCGGGCGCAA//GAGCCCTCCGGGCGGGTCCCCCGGACCCTCCGTTCGAGAGGCTTTGTTAAGCTAGTTATCACCCTGGGCG
PGCI01000623.1_[142074-142831]
CAGTAGCCAACACTTCTAACTTAACACAAGTCCCTCCAATCGGAGGGCCTGGTAACCTGGGCGGGCGCGA//GAGCCCTCCGGGCGGGTCCCCCAGACCCTCCGTTTGAGAGGCTTAGTTAGGCTAGCCAACACTTGGGGTT
PGCI01000644.1_[112313-113050]
ACCCTGAAGGCTTGCCTAACTTAACACAAGTCCCTCCAATCAGAGGGCCTGGTAACCTGGGCGGGCGCAA//GAGCCCTCCGGGCGGGTCCCCCGGACCCTCCGTTCGAGAGGCTTTGTTAAGCTAGAAGGCTTGCAGGCTA
PGCI01000676.1_[72972-73728]
AACACCTCTCTGGCTCTAACTTAACACAAGTCCCTCCAATCGGAGGGCCTGGTAACCTGGGTGGGCGCAA//GAGACCTCCGGGCGGGTCCCCCGGACCCTCCGTTCGAGAGGCTTTGTTAAGCTAGTCTCTGGCTCATCAT
PGCI01000709.1_[18638-19377]
ACCCCCCTAATAGACCTAACTTAACACAAGTCCCTCCAATCAGAGGTCCTGGTAACCTGGGCGGGTGCAA//GAGCCCTCCGGGCGGGTCCCCCGGACCCTCCATTCGAGAGGCTTTGTTAAGCTAGCTAATAGACATGCAT
PGCI01000891.1_[49119-49830]
ACGGGTAGGGTGATGCTAACTTAACACAAGTCCCTCCAATCGGAGGGCCTGGTAGCCTGGCTCGCGGCAA//GAGCCCTCCGGGCGGGTCCCCCAGACCCTCCGTTCAAGAGGCTTTGTTAAGCTAGAGGGTGATGCATCTT

AcademH-N4_PCor
PGCI01000017.1_[132485-133271]
AAATCTAAGAAGGTGCTAATTTAACACAAGCCCTCCTTCTTGGGAGGAGCCGGGACCCCCAGGGAGCGCC//AGTCCTCTTTACAATGGGCCCTCCGGGCCTCCCGGAGGGGACTTAGTTAAGTTAGAAGAAGGTGGTAATA
PGCI01000024.1_[329360-328576]
GAAGGTGTCTTTCCTCTAACTTAACACAAGCCCTCCTTCTTGGGAGGAGCCGGGACCCCCGCGGAGCGCC//AGTCCCCTCTACAAGGGGCCCTCCGGGCCTCCCGGAGGGGACTTAGTTAAGTTAGGTCTTTCCTTATCCT
PGCI01000025.1_[62241-63013]
GACCGCAAGTATCTTCTAACTTAACACAAGCCCTCCTCTTGGGAGGAGCCGGGACCCCAGCGGAGCGCCT//GGATTTAAGTCATAAGTCTCCTCTACAAGTCCTGGAGGGGACTTAGCTAAGTTAGAAGTATCTTGCAGGT
PGCI01000034.1_[143009-142215]
AAATATAGCAGAAATCTCACTCAACACAAGCCCTCCTTCTTGGGAGGAGCTGGGACCCCCGTGGAGCCCC//AGTCCCCTCTACAAGGGGCCCTCCGGGCCTCCCGGAGGGGACTTAGTTAAGTTAGAGCAGAAATGTTCTT
PGCI01000164.1_[184911-184123]
TACATATTCAGTGGCCTAACTTAACACAAGCCCTCCTTCTTGGGAGAAGCCGGGAATCCTGATGAGCGCC//ATTACCCTCTACAAGGGGCCCTCTGGGCCTCCGGGAGGGGACTTAGTTAAGTTAGTTCAGTGGCTTGCTT
PGCI01000187.1_[54115-54903]
AGTTTGGGGTTTCATCTAACTTGACACAAGCCCTGCTTCTTGGGAGGAGCCGGGACACCCGCAGAGCGCC//AGTCCCCTCTACGAGGGGCCCTCCGGGCCTCTTGGAGGGGACTTAGTTAAGTTAGGGGTTTCATTGGATT
PGCI01000193.1_[53011-53807]
CTTAACCAAGTCCCCCTAGCTTAACACAAGCCCTCCTTCTTGGGAGGAGCCAGGACCCCCACGGAGTGCC//TACAAGGTCTACGAGGGGCCCTCCGGGCCACCCGGAGGGGACTTAGTTAAGTTAGCAAGTCCCCCCTGTC
PGCI01000245.1_[58894-59679]
CCCAAGGCCCCAACCCTAACTTAACACAAGCCCTCCTTCTTGAGAGGAGCTGGGACCCCCGCAGAGCGCT//AGTCCCCTCTACGAGGGGCCCTCCGGGCCTCCCGGAGGGGACTTAGTTAAGTTAGGCCTAAGCCACTGCC
PGCI01000716.1_[43724-42940]
GAAGGTGTCTTTCCTCTAACTTAACACAAGCCCTCCTTCTTGGGAGGAGCCGGGACCCCCGCAGAGCGCC//AGTCCCCTCTACAAGGGGCCCTCCGGGCCTCCCGGAGGGGACTTAGTTAAGTTAGGTCTTTCCTTATCCT
PGCI01000728.1_[91832-92604]
GACCGCAAGTATCTTCTAACTTAACACAAGCCCTCCTCTTGGGAGGAGCCGGGACCCCAGCGGAGCGCCT//GGATTTAAGTCATAAGTCTCCTCTACAAGTCCTGGAGGGGACTTAGCTAAGTTAGAAGTATCTTGCAGGT
PGCI01000730.1_[74417-75202]
CCCAAGGCCCCAACCCTAACTTAACACAAGCCCTCCTTCTTGAGAGAAGCTGGGACCCCCACAGAGCGCC//AGTCCCCTCTACGAGGGGCCCTCCGGGCCTCCCGGAGGGGACTTAGTTAAGTTAGGCCTAAGGCACTGCC
PGCI01000938.1_[9277-10063]
ACTACCCTGTAGAGGCTAACTTAACACAAGCCCTCCTTCTTGGGAGGAGCCGGGACCCCCGCGGAGCGCC//AGTCCCCTCTACAAGGGGCCCTTCAGGCCTCCCAGAGGGGACTTAGTTAAGTTAGCTGTAGAGGTAGGTA
PGCI01000978.1_[7209-7996]
AAATCTAAGAAGGTGCTAATTTAACACAAGCCCCCCTTCTTGGGAGGAGCCGGGACCCCCAGGGAGCGCC//AGTCCTCTCTACAATGGGCCCTCCGGGCCTCCCGGAGGGGACTTAGTTAAGTTAGAAGAAGGTGGTAATA
PGCI01001353.1_[3096-3892]
CTTAACCAAGTCCCCCTAGCTTAACACAAGCCCTCCTTCTTGGGAGGAGCCAGGACCCCCACGGAGTGCC//TACAAGGTCTACGAGGGGCCCTCCGGGCCACCCGGAGGGGACTTAGTTAAGTTAGCAAGTCCCCCCTGTC

AcademH-N5_PCor
PGCI01000006.1_[119690-119060]
GTACAATAGACCTTTCTAACTTAACTAAGTCTCTCCTTTGGCGGGAGCTTGAGTTTGGCGGGTGGCAAAG//GCTTCGCCACCGCCAAACTCAAGCTCCCGCATAAGGGAGGACTTAGTTAAGTTAGTAGACCTTTGGGGTC
PGCI01000007.1_[570967-570334]
ATACCGGTCATTGAGCTAACTTAACTAAGTCTTTCCTTTGGCGGGAGCTTGAGTTTGGCGGGTGGAGAAG//CTTTGCCACCCGCCAAACTCAAGCTCCCGCATAAGGGAGGACTTTGTTAAGTTAGGTCATTGAGGTTCAT
PGCI01000111.1_[49333-49966]
AGGTTGAAAATTCAGCTAACTTAACTAAGTCTCTCCTTTGGCGGGAGCTTGAGTTTGGTGGGTGGCGAAG//CTTCGCCACCCGCCAAACTCAAGCTCTTGCATAAGGGAGGACTTATTTAAGTTAGAAAATTCAGGTGTCT
PGCI01000828.1_[74579-73954]
GTACAATAGACCTTTCTAACTTAACTAAGTCTCTCCTTTGGCGGGAGCTTGAGTTTGGCGGGTGGCAAAG//GCTTCGCCACCGCCAAACTCAAGCTCCCGCATAAGGGAGGACTTAGTTAAGTTAGTAGACCTTTGGGGTC
PGCI01000982.1_[20026-20659]
AGGTTGAAAATTCAGCTAACTTAACTAAGTCTCTCCTTTGGCGGGAGCTTGAGTTTGGTGGGTGGCGAAG//CTTCGCCACCCGCCAAACTCAAGCTCTTGCATAAGGGAGGACTTATTTAAGTTAGAAAATTCAGGTGTCT
PGCI01001089.1_[16449-15816]
ATACCGGTCATTGAGCTAACTTAACTAAGTCTCTCCTTTGGCGGGAGCTTGAGTTTGGCGGGTGGAGAAG//CTTTGCCACCCGCCAAACTCAAGCTCCCGCATAAGGGAGGACTTTGTTAAGTTAGGTCATTGAGGTTCAT

AcademH-N6_PCor
PGCI01000008.1_[89787-89027]
GGGGGGCGGCTACGCCTAACTTAACACAAGTCCCAACCGCGGGGGGGGGGGCGGCGTAGCCGCCCTCCTG//GCGCTCCGCGCGGGGTCCCCGGGACCCTCCGTTCGCGAGGCTTATGTTAAGCTAGGGACACGCCCGACCA
PGCI01000025.1_[4704-3952]
TGGCTGGTTGCAAAGCTAACTTAACACAAGTCCCGACCGCGGGGGGGCGGCGTAGCCGCCCTCCCGCAGG//GCGCTCCGCGCGGGGTCCCCGGGATTCTCCGTTTGCGAGGCTTATGTTAAGCTAGGTTGCAAAGCGACCC
PGCI01000055.1_[13765-13011]
CACATGTTGTCATCCCTAACTTAACACAAGTCCCAACCGCGGGGGGGCGGCGTAGCCGCCCCCCCGCAGG//AGCGCTTCGCGCGAGGTCCCGGGACCCTCCGTTCGCGAGGCTTATGTTAAGCTAGTTGTCATCCAATGAA
PGCI01000055.1_[40022-39267]
ACGACCAACTCAACCCTAACTTAACACAAGTCCCGACCGCGGGGGGGGGGGCGTAGCCTCCCTCCTGCAG//GCGCTCCGCGCGGGGTCCCCGGGACCCTCCGTTCGCGAGGCTTATGTTAAGCTATAACTCAACCGATGCA
PGCI01000064.1_[321075-321817]
TTCCTGGCCGCGCGGCTAACTTAACACAAGTCCCGACTGCGGGGGGGCGGCGTAGCCGCCCTCCCGCAGG//CTCCTCCGCCCGGGGTCCCCGGGACCCTCCGTTTGCGAGGCTTATGTTAAGCTAGGCCGCGCGGCATCTG
PGCI01000065.1_[53206-53968]
TTCCTGGCCGCGCAGCTAACTTAACACAAGTCCCGACCTTGGGGGGGGGGGGGCGGCGTAGCCGCCCTC//GCGCTCCGCTTGGGGTCCCCGGAAACCTCCGTTTGCGAGGCTTATGTTAAGCTAGGCCGCGCGGCATCTG
PGCI01000084.1_[286073-285316]
GGGGGGCGGCTACGCCTAACTTAACACAAGTCCCAACCGCGGGGGGGCGGCGTAGCCGCCCTCCTGCAGG//GCGCTCCGCGCGGGGTCCCCGGGACCCTCCGTTCGCGAGGCTTATGTTAAGCTAGGGACACGCCCGACCA
PGCI01000198.1_[21858-21109]
TGGACTCAGCGCAGGCTAACTTAACACAAGTCCCGACCGCGGGGGGGCGGCGTAGCCGCCCTCCCGCAGG//GCGCTCCGCGCGGGGTCCCCAGGAACCTCCGTTCGCGAGGCTTATGTTAAGCTAGCAGCGCAGGACGGTT
PGCI01000590.1_[13595-12841]
CACATGTTGTCATCCCTAACTTAACACAAGTCCCAACCGCGGGGGGCGGCGTAGCCGCCCCCCCGCAGGG//GCGCTTCGCGCGAGGTCCCCGGGACCCTCCGTTCGCGAGGCTTATGTTAAGCTAGTTGTCATCCAATGAA
PGCI01000645.1_[2856-2098]
ACGACCAACTCAACCCTAACTTAACACAAGTCCCAACCGCGGGGGGGGGGGGGGCGTAGCCTCCCTCCTG//GCGCTCCGCGCGGGGTCCCCGGGACCCTCCGTTCGCGAGGCTTATGTTAAGCTATAACTCAACCGATGCA
PGCI01000728.1_[2079-1327]
TGGCTGGTTGCAAAGCTAACTTAACACAAGTCCCGACCGCGGGGGGGCGGCGTAGCCGCCCTCCCGCAGG//GCGCTCCGCGCGGGGTCCCCGGGATTCTCCGTTTGCGAGGCTTATGTTAAGCTAGGTTGCAAAGCGACCC

AcademH-N7_PCor
PGCI01000005.1_[94293-95063]
CACCCACATCAGTCACGAACTTAACACAAGTCCTATCCTTCTCCGGGCGGGGCAGGTTTGGCGGGTCGCA//CCCAGACCTGCCCAACAGGTCCCCCGTCTCACCACAAGAGGCTTAGTTAAGCTCGCATCAGTCAGGGGTT
PGCI01000005.1_[143498-144264]
ATTAGCATATAAGCTCAAACTTAACACAAGTCCTATCCTTCTCTGGGCGGGGCAGGTTTGGCTGGTTGCA//CCCAGACCTGCCCAACAGGTCCCCTGTCTCACCACAAGAGGCTTATTTAAGCTTGATATAAGCTGAAGTC
PGCI01000023.1_[262193-262964]
AATAGAGCTTGGTTACGAACTTAACACAAGTCCTATCCTTCTCCGGGCGGGGCAGGTTTGGCTGGTCGCA//CCCAGACCTGCCCAACAGGTCCCCTGTCTCACCATGAGAGGCTTAGTTAAGCTTGGCTTGGTTAGTAATA
PGCI01000031.1_[277705-278475]
TGCGTGTAAGAATGCCGAACTTAACACAAGTCCTATCCTTCTCCGGGCGGGGCAGGTTTGGCTGGTCGCA//CCCAGACCTGCCCAACAGGTCCCCCGTCTCACCACAAGAGGCTTAGTTAAGCTCGTAAGAATGCCAATTG
PGCI01000060.1_[19124-19891]
CTCTATCCTTGATTCCGAACTTAATACAAGTCCTATCCTTCTCCGGGCGGGGCAGGTTTGGCTGGTCGCG//CCCAGACCTGCCCAACAGGTCCCCCGTCTCATCACAAGAGGCTTAGTTATGCTCGCCTTGATTCAGAGCC
PGCI01000886.1_[15660-16426]
ATTAGCATATAAGCTCAAACTTAACACAAGTCCTATCCTTCTCTGGGCGGGGCAGGTTTGGCTGGTTGCA//CCCAGACCTGCCCAACAGGTCCCCTGTCTCACCACAAGAGGCTTAGTTAAGCTTGATATAAGCTGAAGTC
PGCI01000912.1_[28274-29042]
CTCTATCCTTGATTCCGAACTTAATACAAGTCCTATCCTTCTCCGGGCGGGGCAGGTTTGGCTGGTCGCG//CCCAGACCTGCCCAACAGGTCCCCCGTCTCATCACAAGAGGCTTAGTTATGCTCGCCTTGATTCAGAGCC

AcademH-N8_PCor
PGCI01000002.1_[93364-93493]
CATACGCAGTAATACCGAACTTAACGCAAGCCCCTATTCCCTCTTGTGCGCGGCTTGTAATGCGGTGTGA//TTGTGAGACACAGAGACCCTGCGGGGCTCCTCACCGAGAGGCTTTGTCAGGCTCGCAGTAATACCAATCG
PGCI01000016.1_[12529-12389]
ACTCTAGGGCATTACCGAACTTAACGCAAGCCCCTATTCCCTGCTGTGCGCGGCTTGCAATGCGGTGTGA//GCAGCAGGGACCCTGCGCCTGCGGGGCTCCTCACCGAGAGGCTTTGTTAGGCTCGGGGCATTACTGATTG
PGCI01000018.1_[377712-377574]
GACCTTGCCTCATCGCGAACTCAACGCAAGCCCCTATTCCCTGCTGTGCGCGGCTTGCAATGCGGTGTGA//CTGTGAGACACAGAGACCCTGCGGGGCTCCTCACCGAGAGGCTTTGTTAGGCTTGGCCTCATCGGGGACA
PGCI01000022.1_[521371-521508]
ATGCTGGGCCGCTACCGAACTTAACGCAAGCCCCTATTCCCTGCTGTGCGCGGCTTGCAATGCGGTGTGA//ACCGCAGACACAGAGACCCTGCGGGGCTCCTCACCAAGAGGCTTTGTTAGGCTCGGGCCGCTACAGGTGG
PGCI01000103.1_[175672-175819]
AACCCAGCTTGAGCTCAAACTTAACGCAAGCCCCTATTCCCTGCTGTGCGCGGCTTGCAATGCGGTGTGA//GGGTGAGACACAGAGACCCTGCGGGGCTCCTCACCGAGAGGCTTTGCTAGGCTCGGTTTGAGCTGCTTTC

PGCI01000106.1_[86774-86923]
TGACGGGAATAATCACGAACTTAACGCAAGCCCCTATTCCCTGCTGTGCGCGGCTTGCAATGCGGTGTGA//GGCGCAGGCACAGAGACCCTGCGGGGCTCCTCACCGAGACGCTTTGTTAGGCTCGGAATAATCACAATTA
PGCI01000295.1_[97279-97146]
CTGTGGGCTCCAACACGAACTTAATGCAAGCCCCTATTCCCTGCTGTGCGCGGCTTGCAATGCGGTGTGA//CTGTGAGACACAGAGACCCTGCGGGGCTCCTCACCGAGAGGCTTTGTTAGGCTCGGCTCGAACACAGCCC
PGCI01000609.1_[60613-60742]
CATACGCAGTAATACCGAACTTAACGCAAGCCCCTATTCCCTGCTGTGCGCGGCTTGTAATGCGGTGTGA//TTGTGAGACACAGAGACCCTGCGGGGCTCCTCACCGAGAGGCTTTGTCAGGCTCGCAGTAATACCAATCG
PGCI01000754.1_[13360-13509]
TGACGGGAATAATCACGAACTTAACGCAAGCCCCTATTCCCTGCTGTGCGCGGCTTGCAATGCGGTGTGA//GGCGCAGGCACAGAGACCCTGCGGGGCTCCTCACCGAGACGCTTTGTTAGGCTCGGAATAATCACAATTA
PGCI01000913.1_[32380-32247]
CTGTGGGCTCCAACACGAACTTAATGCAAGCCCCTATTCCCTGCTGTGCGCGGCTTGCAATGCGGTGTGA//CTGTGAGACACAGAGACCCTGCGGGGCTCCTCACCGAGAGGCTTTGTTAGGCTCGGCTCGAACACAGCCC

AcademH-N9_PCor

PGCI01000022.1_[370013-369164]
CACACCTGTCAGTCACAAACTTAACGCAAGCCCCAATTCCCTGCTGTGCGCGGCTTGCAATGTGGTGTGA//CTGTGAGACACAGAGACCCTGCGGGGCTCCACACCGAGAGGCTTTGTTAGGCTCGGTCAGTCACATTTCA
PGCI01000072.1_[286860-287696]
GTGACCAGTGATTTCCAAACTTAACGCAGCCCCTATTCCCTGCTGTGCGCGGCTTGCAATGCGGTGTGAG//TCTGTGATACACAGAGACCCTGCGGGTCTCCTCACCGAGAGGCTTGTTAGGCTTGGTGATTTTCTGATGG
PGCI01000608.1_[127205-126356]
CACACCTGTCAGTCACAAACTTAACGCAAGCCCCAATTCCCTGCTGTGCGCGGCTTGCAATGTGGTGTGA//CTGTGAGACACAGAGACCCTGCGGGGCTCCACACCGAGAGGCTTTGTTAGGCTCGGTCAGTCACATTTCA
PGCI01000862.1_[39982-40830]
TGACCAGTGATTTTCCAAACTTAACGCAAGCCCCTATTCCCTGCTGTGCGCGGCTTGCAATGCGGTGTGA//CTGTGATACACAGAGACCCTGCGGGTCTCCTCACCGAGAGGCTTTGTTAGGCTTGGTGATTTTCTGATGG

AcademH-N10_PCor

PGCI01000067.1_[163440-165620]
AACAGGATTCATACCCTAACTTAACAAAGTCCCTCTGTAACTTCGTGCGGGTCCCAAAGGGCCTCCGAAG//CCTTGGAGGCCCTTTGGGACCCACACGAAGTTACAGAGGGACGTTGTTAAGTTAGATTCATACCCTTGTT

AcademH-N11_PCor

PGCI01000010.1_[26631-25284]
TACATGGACTGGGGACTAACTTGACACAAGTCCTTGGGGGCTGCTGCGACTGCCGCTTGCGGGTCCCCCC//GCGCTTTGTGGGGGGTCCCCGGGACCCTCCGTTTGCAAGGCTTATGTTAAGCTAGGACTGGGGAAGGGAG
PGCI01000012.1_[125430-124059]
TCACCTGGTCTAGATCTAACTTGACACAAGTCCTTGGGGGCTGCTGCGCTGTGTGAGACTGCCGCTTGCG//GCGCTCCGCGCGGGGTCCCCGGGACCCTCCGTTCGCGAGGCTTATGTTAAGCTAGGGTCTAGATGGTGTG
PGCI01000030.1_[264254-265627]
TCCAATCATGCAACTCTAACTTGACACAAGTCCTTGGGGGCTGCTGCGCTGTGTGACACTGCCGCTTGCG//GCGCTCCGCGCGGGGTCCCCGGGACCCTCCGTTTGCGAGGCTTATGTTAAGCTAGCATGCAACTGTACAG
PGCI01000046.1_[295360-296725]
GACAAGCAGTGGATCCTAACTTGACACAAGTCCTTGGGGGCTGCTGCGCTGTGTGACACTGCCGCTTGCA//GCGCTCTGCGCGGGGTCCCCGGGACCCTCCGTTCGCGAGGCTTATGTTAAGCTAGCAGTGGATCCAACGG
PGCI01000628.1_[100257-98885]
TCACCTGGTCTAGATCTAACTTGACACAAGTCCTTGGGGGCTGCTGCGCTGTGTGAGACTGCCGCTTACG//GCGCTCCGCGCGGGGTCCCCGGGACCCTCCGTTCGCGAGGCTTATGTTAAGCTAGGGTCTAGATGGTGTG
PGCI01000815.1_[12455-13820]
GACAAGCAGTGGATCCTAACTTGATACAAGTCCTTGGGGGCTGCTGCGCTGTGTGACACTGCCGCTTGCG//GCGCTCCGCGCGGGGTCCCCGGGACCCTCCGTTCGCGAGGCTTATGTTAAGCTAGCAGTGGATCCAACGG

AcademH-N12_PCor

PGCI01000106.1_[172665-173449]
TAATGTGGTCCTGCCCTAGCTTATCACAAGCCTCTGGTCCTGCGCTTCAGCTGGCCCCGCGGGGCGCGGG//GCGGGGACGCTGGGCCCTTTGGGCCCGCTCCGCTTGAGAGGCTTAGTTATGCTTGGGTCCTGCCTATGTA
PGCI01000191.1_[1953-2750]
TTTATATATCTTCTCCTAGCTTATCACAAGCCTCTGGTCCTGCGCTTCAGCGGACCGCGTGGGGCGCGGG//GTGGGGACGCCGGGCCCTTTGGGCCTGCTCCAATCAAGAGGCTTAGTTAAGCTTGTATATCTTCATGTTG

AcademH-N13_PCor

PGCI01000009.1_[611668-608324]
ATACTATACACACCCCGAACTTAACGCAAGCCCCTTTTCCCTGCTGTGAGCGGCTTGCAATGCGGTATGG//GACCCTGCAGGGCTGCCCCCCAGGGGCTCTCACTCAAGAGGCTTTGTTAAGCTTGTACACACCCACCACC
PGCI01000643.1_[56889-53550]
ATACTATACACACCCCGAACTTAACGCAAGCCCCTTTTCCCTGCTGTGAGCGGCTTGCAATGCGGTATGG//GACCCTGCAGGGCTGCCCCCCAGGGGCTCTCACTCAAGAGGCTTTGTTAAGCTTGTACACACCCACCACC

Figure S2. Termini and TSDs of newly characterized families of *Academ* from two animal species, *Crassostrea virginica* and *Acropora digitifera*. TSDs are colored in red. Positions of TEs are shown in parentheses. Up to 10 sequences are shown for each family.

Crassostrea virginica (Eastern oyster)

AcademH-1_CVi
```
CM008241.1_[12691601-12682832]
GTTGCTGTAACGACCCAATCTAATTAAAATTAGCTGTTTTGATCCGCTACCGCTATTCAAACATCTAACA//TGTTAGATGTTTGAATAGCGGTAGCGGATCAAAACAGCTAATTTTAATTAGATTGTGTAACGACCAAAGG
CM008242.1_[46429846-46438629]
CGTTTGACAGGAAGCCAATCTAATTAAAATTAGCTGTTTTGATCCGCTACCGCTATTCAAACATCTAACA//TGTTAGATGTTTGAATAGCGGTAGCGGATCAAAACAGCTAATTTTAATTAGATTGACAGGAAGCTATCAC
```

AcademH-2_CVi
```
CM008246.1_[19543111-19534916]
AATAATCCATTCATACAATCTGATTAAAATCAGCTGTTCAATATACGCTACCGCTATGAACATCTGAAAC//GTTTCAGATGTTCATAGCGGTAGCGTATATTGAACAGCTGATTTTAATCAGATTGTCCATTCATATACAT
CM008246.1_[29069771-29061660]
TTTCAACCAAACTTGCAATCTGATTAAAATCAGCTGTTTAATATACGCTACCGCTATGAACATCTGAAAC//GTTTCAGATGTTCATAGCGGTAGCGTATATTGAACAGCTGATTTTAATCAGATTGCCAAACTTGGAACAA
CM008248.1_[5687448-5695594]
GGCGTCCAATTTTATCAATCTGATTAAAATCAGCTGTTCAATATACGCTACCGCTATGAACATCTGAAAC//GTTTCAGATGTTCATAGCGGTAGCGTATATTGAACAGCTGATTTTAATCAGATTGCAATTTTATGTGCTG
```

AcademH-2N1_CVi
```
CM008241.1_[231971-232053]
TTGACTGCGTCACTGCAATCTGATTAAAATCACCTGTTCAATATACGCTACCGCTATGAAC-------------------------------GGTAGCGTATTGAACAGCTGATTTTAATCAGATTGTCCATTACTGTTAGT
CM008241.1_[9152003-9152089]
ATATTATGCACATGCCAATTTGATTAAAATCAACTGTTCAATATACGCTACCGCTATGAAC---------------------------GAACGGTAGCGTATTGAATAGCTGATTTTAATCAGATTGTGCACATGCAAATTT
CM008241.1_[28809995-28809911]
CTGCCGTCCAAATCTCAATCTGATTAAAATCAGCTGTTCAATATACGCTACCGCTATG-----------------------------ATAGCGGTAGCGTATATTGAACAGCTGATTTTAATCAGATTGGTCCAAATCTCTATG
CM008241.1_[36739039-36738952]
AAATATTAACAACACCAATCTGATTAAAGTCAGCTGTTCAATGTACGCTACCGCTATG------------------------TTCATAGAGGTAGCGTACATTGAACAGCTGATTTTAATAAGATTGTTTACAACACACGCT
CM008241.1_[39148041-39148123]
TAACAGAAAAATGTTCAATCTGATTAAAATCAGCTGTTCAATATACGCTACTGCTATG--------------------------------AGCGGTAGCGTATATTGAACAGCTGATTTAGTCAGATTGGAAAATGTTTGTTCA
CM008241.1_[55478487-55478422]
AAGGTCTGCTTAGCTCAATCTGATTAAAATCAGCTGTTCAATATACGCTACCGCTATGAAC----------------------------------------------AGCTGATTTTAATCAGATTGGCCTTAGCTCTCTAT
CM008241.1_[64356095-64356035]
AGAATAATCTTTAACCAATCTGATTAAAATCAGCTGTTC-----------------------------------------------------GGTAGCGTATATTGAACAGCTGATTTTAATCAGATTGAATCTTTAACTTTAC
CM008242.1_[8682655-8682580]
AACTGGTCCTTTTCACAATCTGATTAAAATCAGCTGTTCAATATATGCTACCGC--------------------------------------GGTAGCGTATATTGAACAGCTGATTTTAATCAGATTGGTCCTTTTCACAAAA
CM008242.1_[19842042-19842124]
CAACTCCGTCCTGTTCAATCAGATTAAAATCAGCCGTTCAATATACGCTACCGCTATGAACA-------------------------------GTAGCGTATATTGAACAGCTGATTTTAATCGGATTGCATCCTGTTGAACGG
CM008242.1_[21637666-21637579]
AAGTCCTATTTGCCTCAATCTGATTAAAATCAGCTGTTCAATATACGCTACCGCTATGTTCA--------------------------CAGCGGTAGCGTATATTGAACAGCTGATTTTAATCAGATTGTTTTTGCCTGATATT
```

AcademH-3_CVi
```
CM008243.1_[16133205-16124198]
AAACACATTGCCATGCAGTCTGATTAAAACCACCCCCCCTTCTCCTTACACTCGTCGGGGTCTGACAGGC//TGCCTGTCAGACCCCGACGAGTGTAAGGAGAAGGGGGGTGGTTTTAATCAGACTGATTGCCATGAAAATA
CM008243.1_[62290150-62281143]
TGGATAACCCGTTCACAGTCTGATTAAAACCACCCCCCTTCTCCTTACACTCGTCGGGGTCTGACAGGCA//TGCCTGTCAGACCCCGACGAGTGTAAGGAGAAGGGGGGTGGTTTTAATCAGACTGACCCGTTCATAACTT
CM008244.1_[4348948-4357953]
ACAAAACCGACGAGTCAGTCTGATTAAAACCACCCCCCTTCTCCTTACACTCGTCGGGGTCTGACAGGCA//TGCCTGTCAGACCCCGACGAGTGTAAGGAGAAGGGGGGTGGTTTTAATCAGACTGCAACAAAACCAACCG
CM008245.1_[7261975-7270980]
TATGAATAATTTTGTCAGTCTGATTAAAACCACCCCCTTCTCCTTACACTCGTCGGGGTCTGACAGGCAT//TGCCTGTCAGACCCCGACGAGTGTAAGGAGAAGGGGGGTGGTTTTAATCAGACTGTAATTTTGTTTACTG
CM008248.1_[28616690-28607715]
CCATTAATGAAGACTCAGTCTGATTAAAACCACCCCCCTTCTCCTTACACTCGTCGGGGTCTGACAGGCA//TGCCTGTCAGACCCCGACGAGTGTAAGGAGAAGGGGGGTGGTTTTAATCAGACTGATGAAGACTATACTG
```

AcademH-4_CVi
```
CM008246.1_[48795927-48804536]
ATATTGTTGATTTGACAATCTGATTAAAATCAGATCCTTTGTTCCGCTCAAGTACATCGCTACACAAGGA//TCCTTGTGTAGCGATGTACTTGAGCGGAACAAAGGATCTGATTTTAATCAGATTGGTTGATTTGAATTCG
CM008249.1_[65331300-65322678]
AGATCATTTCTTAAACAATCTGATTAAAATCAGATCCTTTGTTCCGCTCAAGTACATCGCTACACAAGGA//TCCTTGTGTAGCGATGTACTTGAGCGGAACAAAGGATCTGATTTTAATCAGATTGATTTCTTAAAAGACT
CM008249.1_[103761947-103753327]
GGTTTGGAAGATGACCAATCTGATTAAAATCAGATCCTTTGTTCCGCTCAAGTACATCGCTACACAAGGA//TCCTTGTGTAGCGATGTACTTGAGCGGAACAAAGGATCTGATTTTAATCAGATTGGAAGATGACGTCATT
```

AcademH-5_CVi
```
CM008244.1_[41290266-41281161]
GTGTCAGTGCCGATGCAATCTAATTAAAATTAGATGTTAGATCCGCTCGCATACTCTCTACACTAACATC//GATGTTAGTGTAGAGAGTATGCGAGCGGATCTAACATCTAATTTTAATTAGATTGGTGCCGATGGTTTAT
CM008244.1_[54752784-54761917]
GCTTTTATTTAAGCCCAATCTAATTAAAATTAGATGTTAGATCCGCTCGCATACTCTCTACACTAACATC//GATGTTTGTGTAGCGAATGTGTGAGCGGTTCAAACATCTAATTTTAATTAGATTGATTTAAGCCCGTCTG
CM008247.1_[24068601-24077721]
AGGCATAGATCAGACCAATCTAATTAAAATTAGATGTTAGATCCGCTCGCATACTCTCTACACTAACATC//GATGTTTGTGTAGCGAATGTGTGAGCGGTTCAAACATCTAATTTTAATTAGATTGAGATCAGACAAACCA
```

AcademH-6_CVi
```
CM008241.1_[17057572-17064928]
TTGTCACTACCAAACCATCGTCGGAACCCACGGGATTTCTATCCGCTACACTTCTTTCATCCCGTGGGAG//CTCCCACGGGATGAAAGAAGTGTAGCGGATAGAAATCCCGTGGGTTCCGACGATGCTACCAAACCTACAT
CM008241.1_[40909671-40902326]
GGTTTGAGTCATATACATCGTCGGAACCCACGGGATTTCTATCCGCTACACTTCTTTCATCCCGTGGGAG//CTCCCACGGGATGAAAGAAGTGTAGCGGATAGAAATCCCGTGGGTTCCGACGATGAGTCATATAAAGCCT
CM008245.1_[29899886-29907233]
ATGCGTGTTAATACTCATCGTCGGAACCCACGGGATTTCTATCCGCTACACTTCTTTCATCCCGTGGGAG//CTCCCACGGGATGAAAGAAGTGTAGCGGATAGAAATCCCGTGGGTTCCGACGATGGTTAATACTCAGTTT
CM008245.1_[57710992-57718347]
ACTCCCAGAGATGTACATCGTCGGAACCCACGGGATTTCTATCCGCTACACTTCTTTCATCCCGTGGGAG//CTCCCACGGGATGAAAGAAGTGTAGCGGATAGAAATCCCGTGGGTTCCGACGATGAGAGATGTAGAATTT
```

AcademH-7_CVi
```
CM008249.1_[21478531-21468732]
CAGAGACTTTTTCCTCAATCTAATTAAAATTAGCCCTTTCATCTCGCTCTCCGTTTGTTTTTGTCGCTCG//CGAGCGACAAAAACAAACGGAGAGCGAGATGAAAGGGCTAATTTTAATTAGATTGCTTTTTCCTGAATCC
CM008249.1_[27484947-27495031]
TCATACAGCAAGACCCAATCTAATTAAAATTAGCCCTTTCATCTCGCTCTCCGTTTTGTTTTTGTCGCTC//GAGCGACAAAAACAAAACGGAGAGCGAGATGAAAGGGCTAATTTTAATTAGATTGAGCAAGACCTTACGT
```

AcademH-7N1_CVi
```
CM008241.1_[7846058-7845724]
CTCAGATTGTCTGATCAATCTAATTAAAATTAGCCCTGTTATCTTTTGTCGCTCGTGAAAGGATCTAACG//AGAGCGACAAAAACAAATGGAGAGCAAGATGAAAGGACTAATTTTAATTAGATTGTTTGTCTGATCTACC
CM008241.1_[8647621-8647974]
TGCTGTTAAGGTGATCAATCTAATTAAAATTAGCCCTTTCATCTTGCTCTCTGTTTGTTTTTGTTGCTTG//CGAGCGACAAAAAAAAAGCGGAGAGCGAAATGAAAGGGCTAATTTTAATAGATTGTTAAGGTGATTTAAT
CM008241.1_[8699381-8699733]
GCTTTGGTAGGCACACAATCTAATTCAAATTAGCCCTTTCATCTCGCTCTCCGTTTGTTTTTGTCGCTCG//CGAGCGACAAAAACAAATGGAGAGCGAGATGAAAGGGCTAATTTGAATTAGATTGGTAGGCACAGAAGTT
CM008241.1_[10641987-10642336]
TATCAAGTCCTACATCAATCTAATTAAAATTAGCCCTTTCATCTCGCTCTCTGTTTGTTTTTGTCGCTCG//ACGAGCGACAAAAACAAACGGAGAGCGAGATAAAGGACTAATTTTAACTAGATTGGTCCTACATATATTC
CM008241.1_[11382732-11383083]
TGTATATGCATTTAGCAATCTAATTAAAATTAGCCCTTTTATCTCGCTCTCTGTTTGTTTTTGTCGCTCG//CGAGCGACAAAAACAAACTGAGAGTGAGATGAAATGGCTAATTTTAGTCAGATTGATGCATTTAGTTTCA
CM008241.1_[12580289-12579936]
ACTTAGAAATGATATCAATCTAATTAAAATTAGCCCTTTCATCTCGCTCTCCGTTTGTTTTTGTCGCTCG//CGAGCGACAAAAACAAACGGAGAGCGAGATGAAAGGGCTAATTTTAATTAGATTGAAATGATATCTAATT
CM008241.1_[13098994-13098645]
TTTGTTGTAATGGATCAATCTAATTAAAATTAGCTCTTTCATCTCGCTCTTCGTTTGTTTATGTCGCTCG//TGAGCGATAAAAACAAACGGAGAGCAAGATGAAAGGGCTACTTTTAATTAGATTGGTAATGGATTAGACA
CM008241.1_[13863664-13863998]
CTCAGATTGTCTGATCAATCTAATTAAAATTAGCCCTTTTATCTTTTGTCGCTCGTGAAAGGATCTAACG//CGAGCGACAAAAACAAATGGAGAGCAAGATGAAAGGACTAATTTTAATTAGATTGTTTGTCTGATCTACC
CM008241.1_[16765832-16766156]
CAACAAGAGATGATGCAATCTAATTAAAATTAGCCTCCGTTTGTTTTTGTCGCTCGTGAAAGGATCTAAC//GTTAGATCCTTTCACGAGCGACAAAAAGATGAAAGGGCTAATTTGAATTAGATTGGAGATGAAGCTACCA
CM008241.1_[18327714-18327363]
ATGGACCTATATAATCAATCTAATTAAAATTAGCCCTTTCATCTTGCTCTCCGTTTGTTTTTGTCGTTCG//CGAGCGACAAAAACAAACGAAGAGCGAGATGAAAGGGCTAATTTTAATAAGATTGCTAAATAATTATCTG
```

AcademH-8_CVi
```
CM008249.1_[53828951-53822030]
GAGTTTACCTAAATCCATCGTCGGAACCCACGGTTGATTACGCTTCGTTCCTCTCTCCATCACCCGTGGG//CCCACGGGTGATGGAGAGAGGAACGAAGCGTAATCAACCGTGGGTTCCGACGATGACCTAAATCGTGTCA
```

AcademH-9_CVi

CM008246.1_[49451734-49440680]
TTGGGTGTTTTCAACCAGTCTGATTAAAATCAAACCTCTCACTTGCTCCCGTTTATTGATATGTCGCGCG//CGCGCGACATATCAATAAACGGGAGCAAGTGAGAGGTTTGATTTTAATAAGACTGGTTTTCAACGCACTC
CM008249.1_[19511525-19522267]
CTTTCCATGAAATATCAGTCTGATTAAAATCAAACCTCTCACTCGCTCCCGTTTATTGATATGTTGCGCG//CGCGCGACATATCAATAAACGGGAGCAAGTGAGAGGTTTGATTTTAATCAGACTGTGTTAGGAGCAATAC

AcademH-N1_CVi

CM008241.1_[11804564-11804878]
AGTGCACAATATGTGCAATCTGACTAAAGTCAGACCTCTATTTATGCTATTGCATAGAGGATCTGATAAC//GTTATCAGATCCTCTATGCGATAGCGTAAATAGAGGTCTGACTTTAGTCAGATTGAAAATATGTGCTGAA
CM008241.1_[14314553-14314931]
CCTTTATAGCTTGCTCAATCTGACTGAAGTCAGATCTCTATTTACGCTATCACATAGAGAATCTGATAAA//GTTATCAGATCCTCTATGCGATAGCGTAAATAGAGGTCTGACTTTAGTCAGATTGTAGCTTGCTTTGTTT
CM008241.1_[17752590-17753000]
ATCATATCTTCTTTACAATCTGACTTAAGTCAGACCTCTATTTACGCTATCACATAGAGGATCTGATAAC//GTTATCAGATCCTCTATGCGATAGCGTAAATAGAGGTCTGACTTTAGTCAGATTGATCTTCTTTACATAT
CM008241.1_[22360873-22361285]
ATTCAGGGACATGCCCAATCTGACTAAAGTCAGACCTCTATGTACGCTATCGCATAGAGGATCTGATAAC//GTTATCAGATCCTCTATGCGATAGCGTAAATAGAGGTCTGACTTTAGTAAGATTGGGACATGCCTCATTA
CM008241.1_[24554982-24554572]
CAAAGGAAACATCATCAATCTGACTAAAGTCAGACCTCTATTTACGCTATCACATAGAGGATCTGATAAC//GTTATCAGATCCTCTATGCGCTAGTGTAAATAGAGGTCTGACTTTAGTCAGATTGGAACATCATCAGTTC
CM008241.1_[28823911-28824265]
ACACTGGAGTGTGTACAATCTGACTAAAGTCAGACCTCTATGTACGCTATCGCATAGAGGATCTGATAAC//GTTATCAGATCCTCTATGCGATAGCGTAAATAGAGGTCTGACTTTAGTCAGATTGGAGTGTGTATACATT
CM008241.1_[29432130-29431813]
CTTCCTTTTGTTGCCCAATCTGATTAAAGTCAGACCTCTATTTACGCTATTGCATAGAGGATCTGATAAC//GTTATCAGATCCTCTATGCGATAGCATAAATAGAGATCTGACTTTAGTCAGATTGAATGTTGCCCGACAG
CM008241.1_[34364572-34364885]
ACAAGGAATAGTACTCAATCTGACTAAAGTCAGACCTCTATTTACGCTATCGCATAGAGGATCTGATAAA//GGGAGTTATCAGATCCTCTAAAATCGTAAATAGAGGTCTGACTTTCGTCAGATTGTAAAAGTACTCGGTA
CM008241.1_[40871597-40871196]
TGTAGAGGGGTGACTCAATCTGACTAAAGTCAGACCTCTATCACATAGAGGATCTGATAACTCTCCATTA//GTTATCAGATCCTCTATGAGATAGCGTAAATAGAGGTCTGACTTTAGTCAGATTGAGGGATGACTGCCAT
CM008241.1_[43263482-43263084]
ATTACAGAAATACAACAATCTGACTAAAGTCAGACCTCTATTTACGCTATCGGAGAGGATATGATTACTC//GTAATGGGGAGTTATCAGATCCTCTATGTGATAGCGTCTGACTTAAGTCAGATTGAGAAATACAATATGG

AcademH-N2_CVi

CM008241.1_[3168518-3168742]
ATTAGTAAATACAGTCAATCTAATTAAAATCAGACCTTCGCTCCGCTCACGCTTTCGCTACTTAAGGATC//TCCATTAGTAGCGATCTAAGGAAGCGGATCAACGGATCTAATTTTAATTAAATTGTAAATATAGTATATC
CM008241.1_[7636018-7636201]
TGTCCCACATTAAAACAATCTAATTAAAATTAGATCCTTTGGTCCGCTCCAAAGGATCTAACGAAACCCC//TCCTTTAGTAGCGATCTAAGGGAGCGGATCAAAGTATCTAATTTTAATTAGATTGACATTAAAATTATTT
CM008241.1_[11149665-11149439]
ATGTAAGGTACTGCACAATCTAATTAAAATTAGATCCTTTGATCCGCTCCCTTAGATCGCTACTAAAGGA//TCCTTTAGTAGCGATCTAAGGGAGCGGATCAAAGGATCTAATTTTAATTAGATTGGGTACTGCACAACGC
CM008241.1_[13377796-13378009]
ACATCCCACTTGTATCAATCTAATTAAAATTAGATCCTTTTGTCCACTCCAAAGGATATAACGAAACCCC//ATCCTTTAGTAGCGATCTAAGGGAGCGGATCAAGGATCTAATTTTAATTATATTGCACTTGTATATAACG
CM008241.1_[14901552-14901313]
AGGGCCCCTCCAGTGCAATCTAATTAAAATTAGATCCTTTGATCCGCTCCCTTAGATCGCTACTAAAGGA//TCCTTTAGTAGCGATCTAAGGGAGCGGATCAAAAATTCTAATTTTAATTAGATTGCCTCCAGTGGAACCT
CM008241.1_[16135471-16135243]
TGGACGACGAAGAACCAATCTAATTAAAATTAGATACTTTGGTCCGCTTCCTTAGATCGCTACTAAAGGA//TCCTTTAGTAGCAATCTAAAGGAGCGGATCAAAGGATCTAATTTTAATTAGGTTGAGAAAGAACAAACAG
CM008241.1_[16202399-16202159]
GAGACGACAAAGAACCAATCTAGTTAAAATTAGATCCTATGATCTGCTCCCTTAGATCGCTACTAAAGGA//TCCTTTAGTAGCGATCTAAGGGTGCAGGTCAAAGGATCTAATTTTAATTAGATTGACGAAGAACAGACAA
CM008241.1_[17608721-17608527]
CATCAGGCATAGCCACAATCTAATAAAAATTAGATCCTTTGATCCGAAACCCCATATAAAGGTTAAATCT//TCCTTTAGTAGCGATCTAAGGTAGCAGATCAAAGGATCTAATTTTAATTAGATTGGCATAGCCAGTAAGT
CM008241.1_[20780785-20781011]
AGAACAATCGATAACCAATCTAATTAAAATTAGATCCTTTGTTCCGCTCCCTTAGATCGCTAGAAACCCC//TCCTTTAGCAGCGATCAAAGGGAGCGGATCAACGGATCTAATTTTAATTAGATTGAATCGATAACAGATT
CM008241.1_[21474785-21475012]
GGAAAAAAACTCTTGCAATCTAATTAGAATTATATCCCTTAGATCGCTACTAACGGATCTAACGAAACCC//TCCTTTAGTAGCGATTTAGGGGAGCGGATCAAAGGCCCTAATTTTAATTAGATTGATAGCTCTTGGTGAT

Acropora digitifera (coral)

Academ-1_ADi

DF971251.1_[156959-149048]
CGTTCAATCACAGCTTAGCCTGCGAAAGCATCCGTTTCTCCTTCGCTCTTCGCCGCTGGGGACGTTTCGC//GCGAAACGTCCCCAGCCGCGAAGAGCGAAGGAGAAACGGATGCTTTCGCAGGCTAGTGATGATGTTGCGG
DF970694.1_[1261389-1261514] (very short derivative)
ATGCCGTGGAATAGGTAGCCTGCGAAAGCATCCGTTTCTCCTTCGCTCTTCGCCGCTGGGGACGTTTCGC//GCCAAACGTCCCCAGCGGCGAAGAGCGAAGGAGAAACGGATGCTTTCGCAGGCTATGGAATAGGCTTAGT

Academ-2_ADi

DF970795.1_[793338-799479]
TAGAAACCTTTCCGACAACCTCGTTCCCAGGGTCTTTCGTCTTCCCATAAGAGGGAGTGGTCGAAGAAAG//CTTTCTTCGACCACTCCCTCTTGTGGGAAGACGAAAGACCCTGGGAACGAGGTTGACAGTGAACAGGAAA
DF970942.1_[156280-150139]
TCAGCCAATTCCGTCCAACCTCGTTCCCAGGGTCTTTCGTCTTCCCACAAGAGGGAGTGGTCGAAGAAAG//CTTTCTTCGACCACTCCCTCTTGTGGGAAGACGAAAGACCCTGGGAACGAGGTTGAATTCCGTCAACATG
DF971416.1_[124641-130773]
CCTATGACCAGAGCCCAACCTCGTTCCCAGGGTCTTTCGTCTTCCCACAAGAGGGAGTGGTCGAAGAAAG//CTTTCTTCGACTACTCCCTCTTGTGGGAAGACGAAATACCCTGGGTACGAGGTTGGACCAGAGCCCTGTG

Academ-2N1_ADi

DF970699.1_[1415214-1416421]
ACAATTGGTTGTTTGCAACCTCGTTCCCAGGGTCTTTCATCCTAGGGAAGAAAGACCCTGGTTCAGGCTG//TTTCTTCCCTCGCTCCTTCGGGGTGGGGAGATGAAAGACCCTGGGAACGAGGTTGGGTTGTTTGTTGTTT
DF970718.1_[853337-854542]
TGGCAGAGTGGAGACCAACCTCGTTCCCAGGGTCTTTCATCCGAGGGAAAGACCCTGGTTCAGGCTGGTC//TTTCCTCCCTCGCTCCCTTGGGGTGGGGAGATGAAAGACCCTGGGAACGAGGTTGAGTGGAGACAGAAAA
DF970719.1_[230915-232172]
ATTCATCGAAATCGCCAACTTCGTTCCCAGGTCTTTCATCCTAGGGAAGAAAGACCCTGGTTCGGGCTGG//TTCGTCCGTCGCTCCCTTTGGGTTGTGGAGATAAAAGACCCTGGGAACGAGGTTGCGAAATGGCCTGAAT
DF970728.1_[1049440-1048213]
TTGCAGCCAATGCTCCAACCTCGTTCCCAGGGTCTTTCATCCGAGGGAAGAAAAACCTTGGTTCAGGCTG//TTCTTCCCTTGCTCCCTTTGGGGTGGGGAGATGAAAGACCCTGGGAACGAGGTTGCAAATCCTCTAACTC
DF970742.1_[929623-928394]
ATGGACGAACAGCTTCAACCTCGTTCCCAGGGTCTTTCATCCAAGGGAAGAAAGACCCTGGTTCAGGCTG//TTCTTCCCTCGCTCGCTTTGGGGTGGGGAGATGAAAGACCCTGGGAACAAGGTTGGAACAGCATCAGAAG
DF970742.1_[1005886-1004681]
ATGGAGGAACAGCATCAACCTCGTTCCCAGGGTCTTTCATCCGAGGGAAGAAAGACCCTGGTTCAGGCTG//TTCTTCCCTCGCTCGATTTGGGGCTGGAAGATGAAAGACCCTGGGAACGAGGTTGGAACAGCATCAGAAG
DF970749.1_[111755-112973]
ACACACACTAGTTCTCAACCTCGTTCCCAGGGTCTTTCATCCGAGACCCTGGTTCAGGCTGGTCACGTGC//GACCTTCCCTCGCTCCTTTGGGGTGGGGAGATGAAAAACCCTGGAAACGAGGTTGACTAGTTCTTGGAAA
DF970760.1_[802038-800798]
GGGCTCGCCCAGTGCCAACCTCGTTCCCAGGGTCTTTCATCCTAGGGAAGAAAGACCCTGCTTCAGGCTG//TTCTTCCCTCGCTCCTTTTGGGGTGGGGAGATGAAAGACCCTGGGAACGAGGTTGGCCCAGTGCCGGTTC
DF970844.1_[688394-689653]
GTTCAAAGTTATTCGCAACCTCGTTCCCAGGGTCTTTCATCCGAGGGAAGAAAGACCCTAGTTCAGAATG//TTCTTTCCTCGCTCCCTTTGGGGTAGGATGATGAAAGACCCTGGGAACGAGGTTGAGTTATTGGTCCAAG
DF970876.1_[512730-511476]
ATGCTTGTTTCGTGGCAACCTTGTTCCCAGGGTCTTTCAACCGAGGGAAGAAAGACCCTGGTTCAGGCTG//TTCCTCCCTCGCTTCCTTTAGGGTGGGGAGATGAAAGACCCTGGGAACGAGGTTGGTTTCGTGGGCGAAT

Academ-3_ADi

DF971246.1_[105961-113111]
TCGTCATCTATGCGTTAGTCTCCTTCGCAGCCGTTATTCGGGTCGTCACGCAATGCTCCTCCCCAACTAA//TTAGTTGGGGAGGAGCATTGCGTGACGACCCGAATAACGGCTGCGAAGGAGACTAGATTTTTGCAGGTAG

Academ-3N1_ADi

DF970693.1_[112738-112435]
CAGGGTCTCCGAGGATAGGCTCCTTCGCAGCCGTTATTCAGGTCGTCACGCAATGCTCTTCCCCAACTAA//TTAGTTGGGGAGGAGCGTTACGTGACGACCCTAATAACGGCTGCGAAGGAGACTACTCCGAGGATGGCTT
DF970694.1_[2282719-2283022]
GATCAATGGAAAATCTAGTCTCCTACGCAGCCGTTATTAGGGTCGTCACGCAATGCTCCTCCCCAACTAA//TTAGCTGGGGAGGAACGTTACGTGACGACCCTAATAACGGCTGCGAAGGAGACTATGGAAAATCATTCTG
DF970695.1_[1176006-1176309]
GGAGCCTCCTTAAGGTAGTCTCCTTCGCAGCCGTTATTCGGGTCGTCACGTAACGCTCCTCCACAACTAA//TTAGTTGAGGAGGAGCATTGCGTGACGACCCTAATAACGGCTGCGAAGGAGACTATCCTTAAACTTGCTC
DF970696.1_[1691628-1691326]
CAGCATGTCCCTTGTTAGTCTCCTTCGCGGCCGTTATCCGGGTCGTCACGCAATGCTCCTCCCCAACTAA//TTAGTTGGGGAGGAGCGTTGCGTGACGACCCTAATAACGTCTGTGAAGGAGACTAGTCCTTTCTGGGCCA
DF970700.1_[300786-301088]
TATCCTCTAAACACTTAGTCTGCTTCGCAGCCGTTATTAGGGTCGTCACGCAATGCTCCTCCCCAACTAA//GTTAGAGGGGAGGAGCATTGCGTGACGACCCTAATAACGTCTGCGAAGGAGACTATCTATCACTCGGCAG
DF970705.1_[1244491-1244794]
AAATGACGGTAAGACTAGTCTCCTTCGCAGCCGTTATTAGGGTCGTCACGTAACGTTCCTCCCCAACTAA//TTAGTTGGGGAGGAACGTTACGTGACGACCCTAATAACGGCTGCGAAGGAGAGTACGGTAAGACGGGCTG
DF970708.1_[997898-998183]
AGACTGTATACTCAATAGTCTACTTCGCGGCCGTTATTAGGGTCGTCACGTAACGCTCACTCGAGCTCTG//GTTAGTGGGGAGAGGCATTGTGCGACAACCCTAATAATGGCTGCGAAGGAGACTATATTCTCAAGAGCTC
DF970709.1_[1216078-1216381]
GTGACGCGGTGGGAGTAGTCTCCTTCGCAGCCGTCATTAGGGTCGTCACGTAACGCTCCTCCCCAACTAA//TTAGTTGGGGAGGGGCATTGCGTGACGACCCTAATAACGGCTGCGATGGAGACTACGGTGGGAGGGACTA
DF970723.1_[267704-267402]
TTTCCCTTTTGAGAGTAGTCTCCTTCGCAGCCGTTATTCGGGTCGTCACGCAATGCTCCTCCCCACTAAC//TTAGTTGGGGAGGAGCATTGCGTGACGACCCTAATAACGGCTGGGAAGGAGACTATTTTGTGAGCCGTTC
DF970724.1_[136144-135841]
GAAGAGGGTGTAGTGTAGTCTCCTTCGCAGCCGTTATTCGGGTCGTCACGTAACGCTCCTCCCCAACTAA//TTAGTTGGGGAGGAGCATTGCGTGACGACCCTAATAACGGCTGCGAAGGAGACTACCCCCTAAGACGAAT

AcademH-4_ADi

DF970724.1_[254106-251533]
AAGTCTTACTGGGATCAATCTCGTTCCCAGAGTCTACGTTCCCCTTGTCCAGCGGAACGGGAAACGAGAG//CTCTCGTTTCCCGTTCCGCTGGACAAGGGGAACGTAGACTCTGGGAACGAGATTGTACTGGGATAAACTG

AcademH-5_ADi

DF970732.1_[927621-923917]

GCTGATACCAAGGGTCAATCTCGTTCCCAGAGTCTACGTTCCCCTTGTCCAGCGGAACGGGAAACAAGAG//CTGTTGTTTCTCATTCCGCTGGACAAGGGGAAAGAAGACTCTGGGAACGAGATTGACCAAGGGTCATTTG

DF971507.1_[144673-148380]

CTAATAGATAGCCCTCAATCTCGTTCCCAGAGTCTACGTTCCCCTTTTCCAGCGGAACGGGAAACAAGAG//CTCTTGTTTCCCGTTCCGCTGGACAAGGGGAACGTAGACTCTGGGAACGAGATTGGATAGCCCTGAGCTT

AcademH-6_ADi

DF971819.1_[37557-44713]

AGTGCCATGTTGAGATAGCCTGTGTCACAGCCGCCTCTCCCCGGAAAAAAATCGGAGGAGAGACGTCTGT//ACAGACGTCTCTCCTCTGATTTTTTTCCGGGGAGAGGCGGCTGTGACACAGGCTAGATTTTATTGAAAAC

AcademH-N1_ADi

DF970758.1_[890398-893551]

CACTCTCTCCAGTGCCAATCTCGTTCCCAGAGTCTTCGTTCCCCTTGACCAGCGGGTCGGGTTGCGAGAG//CTCTCGTAACCCGACCCGCTGGTCAAGGGGAACGAAGACTCTGGGAACGAGACTGGCCAGTGTGCCATAT

DF970706.1_[70558-68597]

ATTCAAAGTAGATTCCAATCTCGTTCCCAGAGTCTTCGTTCCCCTTGTCCAGCGGAACGGGAAACAAGAG//CTCTCGTAACCCGACCCGCTGGTCAAGGGGAACGAAGACTCTGGGAACGAGACTGGCCAGTGTGCCATAT

DF970726.1_[826501-816496]

TTTTTGGTTGCGCAGCAATCTCGTTCCCAGAGTCTTCGTTCCCCTTGACCAGCGGGTCGGGTTACGAGAG//CTCTCGTAACCCGACCCGCTGGTCAAGGGGAACGTAGACTCTGGGAACGAGATTGCAAAATTATCTGGTT

DF970816.1_[331620-334613]

GCGCAGCCATAACATCAATCTCGTTCCCAGAGTCTTCGTTCCCCTTGACCAGCGGGTCGGGTTACGAGAG//CTCTTGTTTCCCGTTCCGCTGGACAAGGGGAACGTAGACTCTGGGAACGAGATTGCCATAACATCCGAGG

DF972116.1_[27518-25420]

GAATTACCACCGTGACAATCTCGTTCCCAGAGTCTTCGTTCCCCTTGACCAGCGGGTCGGGTTACGAGAG//CTCTTGTTTCCCGTTCCGCTGGACAAGGGGAACGTAGACTCTGGGAACGAGATTGCCACCGTGAAAGATT

AcademH-N2_ADi

DF970692.1_[1048074-1048168]

CTTGTTACCTGTAGGCAACCTCGTTCCCAGGGTCTCTCTCCTCTGCCTCCATTGTCATTGAGAATCATCGACAATGGAGGCAGAGAAGAGAGACCCTGGGAACGAGGTTGACCTGTGTCATCCCA

DF970696.1_[435186-435094]

TCTCATAGTATGTCACAACCTCGTTCCCAGGGTCTCTCTTCTCTGCCTCCATTGTCGTTGAGAA--AACGACACTGGAGGCAGAGAAGAGAGACCCTGGGAACGAGGTTGAGTATGTCATACTAT

DF970698.1_[1186067-1186161]

GGTATGCATTCTACCCAACCTCGTTCCCAGGGTCTCTCTTCTCTGCCTCCATTGTCGTCTTTTCTCAGCGACAATGGAGGCAGAGAAGAGAAACCCTGGGAACGAGGTTGCATTCTACCTTGGCT

DF970701.1_[158677-158586]

AAAATGAGGTTATTGCAACCTCGTTCCCAGGGTCTCTCTTCTCTGCCTTCTTTGTCGTT---TCTCAACGACAATGGAGGCAGAGAAGAGAGACCCTGGGAACGAGGTTGAGGTTATTGGTCTTC

DF970702.1_[1029376-1029290]

TGATTATTTGCAGGCCAACCTCGTTCCCAGGGTCTCTCTTCTCTGCCTCCATTGTAGTTGAGA--------CAATGGAGGCAGAGAAGAGAGACCCTGGGAACGAGGTTGTTTGCAGGCAAGGAA

DF970703.1_[636595-636508]

AATGTTTCTGTATGCCAACCTCGTTCCCAGGGTCTCTCTTCTCTGCCTCCATTGTCGTTGAGAA-------CAATGGAGGCAGAGAAGAGAGACCCTGGGAACGAGGTTGTCTGTATGCTAGTTT

DF970708.1_[899366-899274]

GGACATGTATGAAGGCAACCTCGTTCCCAGGGTCTCTCTTCTCTGCCTCCATTGTCGTTGAGAA--AACGACAATGGAGGCAGAGAAGAGAGACCCTGGGAACGAGGTTGGTATGAAGGAGGCGA

DF970708.1_[983856-983767]

CAGGTACCCGTAACACAACCTCGTTCCCAGGGTCTCTCTTCTCTGCCTCCATTGTCGTTGAGAA-----AACAGTGGAGGCAGAGAAGAGAGACCCTGGGAACGAGGTTGCCCGTAACATGGAAT

DF970711.1_[672764-672678]

AAACAATATAAGTAGCAACCTCGTTCCCAGGGTCTCTCTTCTCTGCCTCCATTGTCGTTGAGA--------CAATGGAGGCAGAGAAGAGAGACCCTGGGAACGAGGTTGTATAAGTAGTCAATT

DF970719.1_[845600-845513]

AGTGATATTTGGCATCAACCTCGTTCCCAGGGTCTCTCTTCTCTGCCTCCATTGTCGTTGACGA-------CAATGGAGGCAGAGAAGAGAGACCCTGGGAACGAGGTTGATTTGGCATAAATAC

AcademH-N3_ADi

DF970692.1_[2321425-2321521]

TAGCGAGCTAAACGCTAGTCTCCTTCGCAGCCGTTTTTCGGGATGTCACGCAACGCTCCCC----------TTTCGGGGGAGCGTTGCGTGACATCTCGAAAAACGGCTGCGAAGGAGACTAGCTAAACGCCGGCCA

DF970696.1_[1955681-1955596]

AACAATATGGTGTTCTAGTCTCCTTCGCAGCCGCCTTTCGAGATGTCACGCA---------------------GCGGGGGAGCGTTGCGTGACATCCCGAAAGACGGCCGCGAAGGAGACTAATGGTGTTCAATCGA

DF970696.1_[1955810-1955725]

CTGCCTATGGTGTTCTAGTCTCCTTCGCAGCTGTCTTTCGAGATGTCACGCA---------------------GCGGGGGAGCGTTGCGTGACATCCCGAAAGACGGCCGCGAAGGAGACTAATGGTGTTCAATCGA

DF970702.1_[315044-314963]

CTTCATTGCCCTTAATAGTCTCCTTGGCAGCCGTCTTTTGGATGTCACGCAACG------------------------GGAGCGTTGCGTGACATCC-AAAAGACGGCTGCCAAGGAGACTATGCCTTTTCCTATAA

DF970708.1_[704734-704628]

CTTCCCATCATCCCTTAGTCTCCTTCGCAGCCCTCTTTCGGGATGTCACGCAACGCTCCCCCCGTTCCTTCTTTCGGGGGAGCGCTGCGTGACATCCCGAAAGACGGCTGCGAAGGAGACTAATCATCCCTTAAAAT

DF970714.1_[866629-866532]

CGTCCTGATGAACAGTAGTCTCCTTCGCAGCCGTCTTTCGCGATGTCACGCAACGCTCC---------GCCTTTCGGGGGAGCGTTGCGTGACATCCCGAAAGAAGGCTGCGAAAGAGACTAGATGAACAGTAGCGA

DF970722.1_[1034550-1034646]

TTGCCTGGACACAGGTAGTCTCCTTTGCAGCCGTCTTTCAGGATGTCACGCAACATCCCCC----------TTTCGGGGGAGCGTTGCGTGACATCCCGAAAGACGGCTGCGAAGGAGACTACTGGCCGCCCAGGCA

DF970732.1_[295252-295167]

AGGTTTGTAGGAGCCTAGTCTCCTTCACAGCCATCTTTCGGGATGTCACGCAAC---------------------GGGGGAGCGTTGCGTGACATCCCAAAAGACGGCTGCGAAGGAGACTAGTAGGAGCCTGAAGT

DF970733.1_[1073577-1073677]

AATTTAGTGTTTCCTTAGTCTCCTTCGCAGCCGTCTATCGGGATGTCACGCAACGCTCTCCC------GAAAGGTTCCTTTTCGTTGCGTGACATCCCGAAAGACGGCTCCGAAGGAGACTAGTGTTTCCTTTACAA

DF970736.1_[317918-318018]

AAGCTACGTTTTCCTTAGTCTCCTTCGCAGCCGTCTTTCGGGATGTCACGCAACGCTCCCC------GTTTCTTTCGGGGAGCGTTGCGTGACATCCCGAAAGACGGCTGCGAAGGAGACTACGTTTTCCTCTACGT

AcademH-N4_ADi

DF970692.1_[2235390-2235307]_

TGACGAGAGAAGTCTCAACCTCGTTCCCAGGGCCCCTCGTCTTCCCTTCC-----------------------------TCTCTCGCTCCAGGGGAAGGGAAGACGAGAGGCCCTGGGAACGAGGTTGGAGAAGTCTTTGGTT

DF970787.1_[176189-176107]

AAACTGGGTACCACCCAACCTCGTTCCCAGGGTCTCTCATCTTAACGCCTGGGGCGAACGAGGAGA------------------------------AGGGAAGAGGAGAGGCCCTGGGAACGAGGTTGGGTACCACCAGTCCA

DF970792.1_[571694-571806]

TGAGGGAGCTCATCACAACCTCGTTCCCAGGGCCTCTCCTCTTCCTTTCCCCTGGAGCGAGAGAGGAGAGAATATCTCCTCTCTCGCTCCAGGGGAAGGGAAGAGGAGAGGCCCTGGGAACGAGGTTGAGCTCATCACTGTAT

DF970807.1_[449991-449884]

TTTCAAATGCAGCGACAACCTCGTTCCTAGGGCCTCTCCTCTTCCCTTCCCCTGGAGCGAGAGAGGA-----TCTCTCTTCTCTCGCTCCAGGGGAAGGGAAGAGGAGAGGCCCTGGGAACGAGGTTGATGCAGCGACGGCTG

DF970807.1_[605455-605537]

AATCAGACAATTCGGCAACCTTGTTCCCAGGGCCTCTCGTCTTCCCTTCC-----------------------------TCTCTCGCTCCAGGG-AAGGGAAGAGGAGAGGCCCTGAGAACGAGGTTGACAATTCGGCCGCCG

DF970811.1_[741943-741860]

AAAGTACTGGCTTTCCAACCTCGTTCCCAGGGCCTCTCGTCTTCCCTTCC-----------------------------TCTCTCGCTCCAGGGGAAGGGAAGACGAGAGGCCTTGGGAACGAGGTTGCTGGCTTTCTACTTA

DF970819.1_[562263-562179]

AAGCTCTAAAATTAACAACCTCGTTCCCAGGGCCTCTCGTCTTCCCTCTCC----------------------------TCTCTCGCTCCAGGGGAAGGGAAGACGAGAGGCCCTGGGAACGAGGTTGTAAAATTAAAAGGCA

DF970835.1_[423676-423759]

TAAATTCTCGTGATACAACCTCGTTCCCAGGGCCTCTTCTCTTCCCTTCC-----------------------------TCTCTCGCTCCAGGGGAAGGGAAGAGGAATGGCCCTGGGAACGAGGTTGCTCGTGATATTCCTT

DF970839.1_[474078-474161]

ACGCTGTTCAGTGTTCAACCTCATTCCCAGGGCCTCTCCTCTTCCCTTCA-----------------------------CCTGTCGCTCCAGGGGAAGGGAAGAGGAGAGGCCCTGGGAACGAAGTTGTTCAGTGTTTTGCAG

DF970840.1_[494960-495049]

AAGTTATGGCAAGTGCAACCTCATTCCCAGGGCCTCTCCTCTTCCCTTTA-----------------------CCCTCCTCTCTCGCTCCAGGGGAAGGGAAGAGGAGAGGCACTGGGAACAAGGTTGTGGCAAGTGTTCATG

AcademH-N6_ADi

DF970695.1_[554537-554472]

TCAATGTTGTTCTATCAACCTCGATTCCAGGGCTTTTCTCCTT-----------------------CCTCTCATCGGCGGAGAAAAGCCCTGGGAACGAGGTTGTTGTTCTATGAGAAA

DF970697.1_[1083212-1083149]

GTCTCTTTTTATTCTCAACCTCGTTCCCAGGGCATTTCTCCGC-----------------------CTCTTCT-CAACGGAGAAAAGCC-TGGGAACGAGGTTGGTTTGTTCTGCTCCT

DF970702.1_[87740-87656]

GCGTGCCATGACGAGCAACCTCGTTCCCAGGGCTTTTCTCCGCCGAGGAGAGGAA---GGCCATTCCCCTCC--TCGGCGGAGAAAAGCCCTGGGAACGAGGTTGCCATGACGAGTATAC

DF970707.1_[637769-637680]

TCCCTATGACCCGAACAACCTCGTTCACAGGGCTTTACTCCGCCGAGGAGAAGGGCTTTCCATCCGCCCTCTCGTCGGCGGAGAAAAGCCCTGGGAACGAGATTGTGACCTGTAGCCTCT

DF970728.1_[241489-241404]

AAATAGAAAGGTTTACAGCCTCGTTCCCAGGGCTTTTCTCCGCCGAGGAGAGGATGGGCCCATCCTCT--CC--TCGGCGGAGAAAAGCCCTGGGAACGAGGTTGAAAGATTTACAGATG

DF970732.1_[539178-539102]

GGAGACAATTTATAGCAACCTCGTTCCCAGGGCTTTTATCCGACGAGGAGAGGAAGGGCCC-------------TCGGCGGAGAAAAGCGCTGGGAACGAGGTTGAATTTATAGATGTCG

DF970735.1_[194895-194826]

ACATGGGAAGACCTTCAACCTCGTTCCCAGGGCTTTTCTCCGCCGAGGAGAGGATGGG--------------------CGGAGAAAAGCCCTGGGAACGATGTTGGAAGACCTTAACTAA

DF970741.1_[103115-103055]

AAGGCTAGGCAGCGGCAACCTCGTTCCCAGGACTTCTCTCCTT-----------------------------CCTCGGCGGCGAAAAGCCCTGGGAACGAGGTTGAGGCAGAGGGAGACT

DF970743.1_[539635-539556]

AAAACTAATAAAAGGCAACCTCGTTCCCAGGGCTTTTCTCCGCCGAGGAGAAGA----------TGGCCTCTCCTCGGCAGAGAAAAGCCCTGGGAACGAGGTTGAATAAAAGGTACAGT

DF970766.1_[388094-388025]

CTTAAGCGGGAAAGACAACCTCGTTCCCAGGGCTTTTCTCCGCTCGT--------------------CCTCTCCTCGGCGGAGAAAAGCCCTGGGAACGAGGTTGGGGGAAAGATACTTA

AcademH-N7_ADi

DF970693.1_[1194374-1194018]

TTACCTCATCGAGACCAACCTCGTTTCAAGGGCTTATTCCATAAGAAAGTGGGAAGGGCGGGAAAAGGCC//GGGTCTTTTCCCGCCCTCCCATTTTCTTAGGGAAAAAGCCCCGGGAACGAGGTTGCATCGAGACGAAACG

DF970733.1_[408012-408373]

CATGTGCTTGCATGACAACCTCGTTCCTAGGGCTTTTTTTCCTAATAAAATGGGAAGGAAGGGAAAAGGC//GGCCTTTTTCCGCCCTTCCCATTTTCTTAAGGAAAAAGCCCTGGGAACAAGGTTGCTTGCGTGAGGCACC
DF970746.1_[197216-197510]
AAGCACGTCGATTAACAACCTGTTCCCAGGGCTTTTTCCTAAGAAAATGGGAAGGGTGGGAAAAGGCAC//GGCCTTTTCCCGCCCGTCCTATTTTCTTAGGGAAAAAGTCCTCGGAACGAGGTTGGTCGATCAAAGTTGC
DF970746.1_[471736-472103]
AAGCACGTCGATTAACAACCTCGTTCGCAGGGCTTTTTCCCTAAGAAAATGGCAAGGGCGGGAAAAGGCA//TCCCGCCCGTCCCATTTTCTTAGGGAAAAAGCCCTGGGATCCCGGAACGAGGTTGGTCGATTAAAGTTGC
DF970760.1_[359889-359601]
AAGCACGTCGATTAACAACCTCGTTCCCAGGGCTTTTTTCCTAAGAAAATGGGAAGGGCGGGAAAAGGCA//GGCCTTTTCCCGCCCGTCTTATTTTCTTAGGGAAAAAGCCCTGGGAACGAGGTTGGTCGATCAAAGTTGC
DF970778.1_[852370-852729]
AAGCACGTCGATTAACAACCTCGTTCTCAGGGCTTTTTTCCTAAGAAAATGGGAAGGGCGGGAAAAGGCA//GGCCTTTTCCTGCCCGTCCTATTTTCTTAGGGAAAAAGCCCTGGGAACGAGGTTGGTCGATCAAAGTTGC
DF970833.1_[250048-249760]
AAGCACGTCGATTGACAACCTCGTTGCCAGGGCTTTTTTCCTAAGAAAATGGGAAGGACGGGAAAAGGCA//GGCCTTTTCCCGCCCGTCCTTTTTTCTTAGGGAAAAAGCCCTGGGAACGAGGTTGGTCGATCAAAGTTGC
DF970839.1_[576106-575654]
GATACGCGGTTTGAGCAACCTCGTTCCCAGCGCTTTTTCCTTAAGAAAATGAGAAGGGCGGGAAAAGGCC//GGCCTTTTCCTGCCCTTCCCATTTTCTTAGGGAAAAAGCCCTGGGAACGAGGTTGGCGGTTTGAGTTATC
DF971019.1_[343102-343390]
AAGCACGTCGATTAACAACCTCGTTCCCACGGCTTTTTTCCTAAGAAAATGGAAAGGGAGGGAAAAGGCA//GGCCTTTTCCCGCCCGTCCTATTTGCTTAGGGAAAAAGCCCTGGGAACGAGGTTGGTCGATCAAAGTTGC
DF971113.1_[305228-305591]
AGTTAACTGACCCAGCAACCTTGTTCTCAGGGCTTTTTCCCTAAGAAAATGGGAAGGGCGGGGAAAGGCC//GGGCCTTTTCCCTCCCTCCCATTTTCTTAGGGAAAAGGCCCTGGGAACGAGGTTGCTGACCCAGCTCACT

AcademH-N8_ADi

DF970737.1_[1103503-1105053]
TAGACTAACTGGCGTTAGCCTGCGAGCAGGCTCTTCCGTTGAAGGGCTTGGTAACTAGTCAAGAGAAGAA//TTCTTCTCTTGACTAGTTACCAAGCCCTTCAACTGAAGAGCCTGCTCGCAGGCTAAACTGGCGTGGGGTT
DF971536.1_[82999-81410]
GACCTTATCTGCGGCTAGCCTGCGAGCAGACTCTTCCGTTGAAGGCCTTGGTAACTAGTCAAGAGAAGAA//TTCTTCTCTTGACTAGTTACCAAGGCCTTCAACGGAAGAGCCTGCTCGCAGGCTAATCTGCGGCTGTTTA

AcademH-N9_ADi

DF970955.1_[19560-20127]
AAGAGGCTATATAGTCAATCTCGTTCCCACAGCCTCCACTACCCTTGTCCAGCGGAACGGGCGCAGGAGA//AGTCTCTCTTTCCCGACTTCTGACCAAGGGAACGATGACTTTGGGAACGAGATTGCTATATAGTGGCCTT
DF970955.1_[186810-187377]
AAGAGGCTATATAGTCAATCTCGTTCCCACAGCCTCCACTACCCTTGTCCAGCGGAACGGGCGCAGGAGA//AGTCTCTCTTTCCCGACTTCTGACCAAGGGAACGATGACTTTGGGAACGAGATTGCTATATAGTGGCCTT

AcademH-N10_ADi

DF970709.1_[957221-955451]
GGATAGGTCGCCTTTCAACCTCGTTCCCAGGGTCTACTCCGCTTTCAAGATGGCGCCCGAGAGAAGACCC//GGGTCTTCTCTCGGTCGCCATCTTGAAAGCGGAGTACACCCTGGGAACGAGGTTGGTCGCCGTTAAGGTG
DF970721.1_[47499-49689]
TCCGCATATCACTTGCAACCTCGTTCCCAGGGTCTACTCCACTTTCAAGATGGCGGCCGAGAGAAGACCC//GGGTCTTCTCTCGGCCGCCATCTTGAAAGTGGAGTATACCCTGGGAACGAGGTTGTATCACTTGCTACTT
DF971341.1_[22920-21224]
ACAATACATTGCAGTCAACCTCGTTCCCAGGGTCTACTCCGCTTTCAAGATGGCGACTGAGAGAAGACCC//GGGTCTTCTCTCAGCCGCCATCTTGAAAGCGGAGTAGACCCTGGGAACGAGGTTGCATTGCCACTTTTTC
DF971787.1_[74928-73231]
GATTCACGGACTTCCCAACCTCGTTCCCAGGGTCTACTCCGCTTTCAAGATGGCGGCTGAGAGAAGACCC//GGGTCTTCTCTTGGCCGCCATCTTGAAAGCGGAGTAGACCCTGGGAACGAGGTTGCTATCAAAATAAAAT

AcademH-N11_ADi

DF970810.1_[114391-115455]
GAGTTCAACTCCGGCTACCCTGAGTGCCAGATGGTTTTTTCCTTTGTCGTGATCTATAAGATACCGAGCC//GTTTTAACCGCTCGCTCCTCGCGAAAGAAAAATAGAACCTCTGGCACCCAGGGTAAACTCCGGCCGGACC
DF970882.1_[502964-504112]
ACACATGCAGAACTTTACCCTGGGTGCCAGAGAGTTTTTTTCCTTTGGCGTGATCTAGCTATAAGATAC//GTTTTAACCGCTCGCTACTCGTGAAAGAGAAATAGAACCTCTGGCACCCAGGGTACCATCATGTTATTTG
DF970979.1_[322944-324064]
TAAGAACAAAAAATATACCCTGGGTGCCAGAGGGTTTTTTTCCTTTGGCGTGATCTAGCTATAAGATAC//GTTTTAACCGCTCGCTCCTCGTGAAAGAAAAATAGAATCTCTGGCACCCAGGGTACAAAAAATAGGGAGC
DF971042.1_[352889-351779]
TACTGGCCATCAAGCTACCCTGGGTGCCAGGGTTTTTTTTCCTTTGCCGTGATCTATAAGATACAAAGC//GTTTTAACCACTCGCTCCTCGAGAAAGAAAAATAGAACCTCTGGCACCCAGGGTAGCGTCAAGTCTAATT
DF971521.1_[122581-121435]
AAGCCAAATCTCAACTACCCTGGGTGCCAGAGAGTTTTTTTTTTCCTTTGGCGTGATCTAGCTATAAGA//GTTTTAACCGCTCGCTCCTCGTGAAAGAAAGATAGAACCTCTGGCACCCAGGGTAAATCTCAACGTGCAA
DF971729.1_[18943-17804]
CCTCAACCTATATTCTACCCTGGGTGCCAGAGTGTTTTTTTTCCTTTGGCGTGAGCTAGCTATAAGATAC//CGTTTTAACCGCTGCTCCTCTTGAATGAAAAATAGAACCTCTGGCACCTAGGGTACATATATTCTTGAGT
DF972228.1_[26002-24936]
TAATGGCCGTCAAGCTACCCTGGGTGCCGGAGGGTTTTTTTTCCCTTTGGCGTGATCTATAAGATACCG//GTTTTAACCGCTCGCTCCTCGAGAAAGAAAAATAGAACCTCTGGCACCCAGGGTACCGTCAAGCCTAATT
DF972266.1_[13982-15050]
TAGATAGGGTGCTCTTACCCTGGGTGCCAGAGGGCTTTTTCCTTTGGCGTGATCTAGCTATAAGATACC//GTTTTAACCGCTCGCTCCTCGTGAAAGAAAAATAGAAGCTCTGGCACGCAGGGTAGGGTGCTCTCGAACC

AcademH-N12_ADi

DF970737.1_[180591-179039]
GCAATGCGGTTTAGTTAGCCTGCGAGCAAGCTCTCTCGTGCGGTGTGGGTGGGTGGAGAGAGGAAGAGAG//CGCTCTTCCTCTCTCCACCCACCCACACTGCACGAGAGAGCTTGCTCGCAGGCTACGGTTTAGTTCCACA
DF970749.1_[780974-782521]
CCCTGGTTTAATCCCTAGCCTGCGAGCAAGCTCTCTCGTGTGGTGTGGGTGGGTGGAGAGAGGAAGAGAG//CTCTCTTCCTCTCTCCACCCACCCACACCGCACAAGAGAGCTTGCTCGCAGGCTATGTAAAAGCAAGTGC
DF971749.1_[25856-24318]
CCCCGGTTTAATCCCTAGTCTGCGAGCAAGCTCTCTCGTGCGGTGTGGGTGGGTGGAGAAAGGAAGAGAG//CTCTCTTCCTCTCTCCACCCACCCACACCGCACGAGAGAGCTTGCTCGCAGGCTACCATATTATTATTTA
DF971631.1_[43387-44937]
NNNNNNNNNNNNNNNTAGCCTGCGAGCAAGCTCTCTCGTGCGGTGTGGGTGGGTGGAGAGAGGAAGAGAG//CTCTCTTCCTCTCTCCACCCACCCACACCGCACAAGAGAGCTTGCTCGCAGGCTAATAAAATTAGTGCGC

AcademH-N14_ADi

DF970857.1_[396772-395593]
TATCCCATATTGTTATAGACTGCGTGCAGTCCTTCTTCGGTCAGTCACGTCTAAGTTCGGCAGGACTGGA//TCCAGTCCTGCCGAGATCAGACGTGACTGACCGAAGAGGGACTGCTCGCAGTCTAACATTGTTAAGTGCC
DF970880.1_[552637-553747]
GGGGGGGGGGGGGGTTAGACTTCGAGCAGTCCCTCTTCGGTCAGTCACGTCTAAGTTCGGCAGGACTGGA//TCCAGTCCCGCCGAGCTTAGACATGACTGAGCGAAGAGGGACTGCTCGCAGTCTAGGGGGGGGTTGCCTC
DF970924.1_[445447-444338]
TCACTCAAACCTCCCTAGACTGCGACCAGTCCCTCTTCGGTCAGTCACGTCTAAGTTCGGCAAGACTGGA//TCCAGTCCTGCCGAGCTTAGACGTGACTGACCGAAGAGGGACTGCTCGCAGTCTAAAACCTCCCTATCAA
DF971197.1_[21560-20509]
CAAACACCATATTCCTAGACTGCGAGCAGTCCCTCTTCGGTCAGTCACGTCTAAGTTCGGCAGGACTGGA//TCCAGTCCTGCCGAGATTAGACGTGACTGACCGAAGAGGGACTGCTCGCAGTCTACCATATTCCTTAAAA
DF971525.1_[58401-59517]
TTTGAAGGGCATTCCTAGACTGCGAGCAGTCCCTCTTCGGTCAGTCACGTCTAAGCTCGGCAGGACTGAA//TCCATTCCTGCCGAGCTTAGACGTGACTGACCGAAGAGGGACTGCTCGCAGTCTAGGGCATTCCCGACCG
DF971603.1_[37293-36229]
TTTCTCTTGTTTTCATAGACTGCGAGCAGTCCCTCTTCGGTCAGTCACGTCTAAGTTCGGCAGGACTGGA//TCCAGTCCTGCCGAGATTAGACGTGACTGACCGAAGAGGGACTGCTCGCAGTCTATTGTTTTCATGTCTA

Academ-4_ADi

DF970725.1_[858884-862250]
ATCGCACGAGCCTTTTTGTCCGGGAGCAAATGTCATAAAATAAGGTCCCGTGGGATTTTTTTGCTGAAAG//CAGTGGCTTGCTTTCAGCACAAACTTCCCACGGGAGTACACTTGCTCCCAGACTAGAAGTCGGGCGCGCG
DF971040.1_[231320-221366]
GAAAGCTCGTTATACTAGTCCGGGAGCAAATGTCATAATACATGGTCCCGTGGAATTTTTTTGCTGAAAG//CAGTGGCTTGCTTTCTGCACAAACTTCCCACGGGAGTACACTTGCTCCCTGACTATACGTTCCATTTCTC
DF971227.1_[99230-108793]
GATTTGTCTCCGATATAGTCCGGGAACAAATGTCATAATATAAGGTCCCGTGCGATTTTTTTGCTGAAGG//CAGTGGCTTGCTTTCAGCACAAACTTCCCACGGGAGTACACTTGCTCCCAGAGTAATATAGGGTGGTGCA
DF971231.1_[84451-82349]
CTCCGATTGCCGTTATAGTCCAGGAGCAAATGTCATAATATAAGGTCCCGCGGGACTTTTTTGCTGAAAG//CAGTGGCCTGCTTTCAGCACAAACTTCTCACGGGAGTACACTTGCTCCCAGACTATTACCCTCTGCATCC
DF971354.1_[132278-129290]
GGATTCCACTCAGTCTAGTCCGGGAGCAAATGTCATAATATAAGGTCCCGTGGGATTTTTTTGCTGAAAG//CAGTGGCTTGCTTTCAGCACAAACTTCCCACGGAAGTACACTTGCTCCCAGACTAGTCCTTTTACAATTA
DF971568.1_[90108-92428]
CTCCCTGACTATTAATAGTCCGGGAGCAAATCTCATAATATAAGGTCCCGTGGTATTTTTTTGCTGAAAG//TAGTGGCTTGCTTTCAGCACAAACTTCCTACGGGAGTACACTTGCTCCCAGACTATAATAGAGAAGTACA
DF971930.1_[12724-9543]
GGTAGGAGATAGATTTAGTCTGGGAGCAAATGTCACAATATAAGGTCCCGTGGGATTTTTTTGCTGAAAG//CAGTGGCTTGCTTTCAGCACAAACTTCCCACGGGAGTACACTTGCTCCCAGACTAATTTGCTTGTAAGAA

Academ-5_ADi

DF971126.1_[325442-322036]
AAGTATTAGTCTAATTAGTCTGGCAGCATGTGTAACAAATTAAAGTCCCGTGGGCATTTCGTAAAGAAAA//AGGAAGCATGTTTTCTTCACGAAATGCCCACGGAATTACCAATGCTGCCAGACTAAATGAAGGCGGGTCA

Academ-6_ADi

DF971274.1_[85464-88370]
ATTCCGTAAGGGTTATAGTCCAGTCATTTTATGGAAAAAATCGGTCAACCTGCAACTAATTGCACCAGAA//AGAGAAAGGTTCTGTTGCAATTAGTGGCAGGTCCAGCCACAAAATGACTGGACTATTAACAGAGGCGAGT

Academ-7_ADi

DF970909.1_[471096-474588]
GAAAACGGCAATAAATAGTCCAGGCATTTTATTGACAAAAGTCGGCCAACCTGCCACTAATTGCAAAAGA//TGAGAAATCTTCTTTTGCAATTAGTTGCAGGTCCCTCGACAAAATGCCTGGACTAAAAAACAAGACAAAT
DF971017.1_[377178-379783]
TCTAAATAGGCATATTAGTCCAGGCATTTTATCGTCAAAAGTCGGCCAACCTGCCACTAATTGCAAAAGA//TGAGAAAGCTTCTTTTGCAATTAGTTGCAGGTCCCTCGACAAAATGCCTGGACTATATAATTGGTCATAG
DF971063.1_[137840-134472]
GTTGATTTCTACTTATAGTCCAGGCATTTTATCGTCAAAAGTCGGCCAACCTGCCACTAATTGCAAAAGA//TGAGAAAGCTTCTTTTGCAATTAGTTGCAGGTCCCTCGACAAAATGCCTTGGCTATTACTGTACTTCGAG

Academ-8_ADi

DF970694.1_[190177-195386]
TAATTATTCTTAGTATAGTCAGGGGGCCAGACTCCTCTATCATGCTTAAAAATGGCTTTGACATGGACGG//TTTATATCAAGGTGTCCATCATTTCGCAAAAATTTGGGCTCTGGCCCCCTGAATAGTAGCGCATGTGAAC
DF970782.1_[65078-69838]
AAACAAAATTAATTATAGTCAGGGGGCCAGACTCCTCTATCATGCTTAAAAATGGTCTTGACATGGACGG//TTTATATCAAGGTGTGCATCATTTCGCAAAAATTTGGGCTCTGGCCCCCTGACTATTAGGGGTTGAAAAT
DF971552.1_[24861-31395]
TGTCACCTGTACTACTAGTCAGGGGGCCAGACTCCTCTATCATGCTTAAAAATGGCCTTGATATGGACGG//TTTATATCAAGGTGTCCATCATTTCGCAAAAAATTGGGCTCTGGCCCCCTGACTATACGTAATCACTAGC
DF971552.1_[107010-113544]
TGTCACCTGTACTACTAGTCAGGGGGCCAGACTCCTCTATCATGCTTAAAAATGGCCTTGATATGGACGG//TTTATATCAAGGTGTCCATCATTTCGCAAAAAATTGGGCTCTGGCCCCCTGACTATACGTAATCACTAGC

Academ-9_ADi

DF971075.1_[195335-193449]
ACACGCCACCACTTATAGTCCATGGGCCAGACCACTTTTCGGTACCAAACATAAGGACAAAAACTGAGTG//TTATCCCTCGTTTTGGTACCAAATAGCTCTTTTTAGGGTCCTGGCCCATGGACTATTACAGCGGTTTCTT

Academ-N1_ADi

DF971183.1_[56453-58977]
TTCTTTCCCGTGTTTTAGACTGGGACAAATTCCAAGAAAGCCTGCTATTTTAAACTTCTGTCGGCAAACT//ACTTTTCCTCGAGTTTGCCGACAGAAGTCCACTTTTTGCGAAATGTCCCAGTCTATTTTGCAACATTAAA

Academ-N2_ADi

DF971375.1_[158936-156240]
ATAAATAAACTCAAATAGTCAGGCGGTTATTTGCAGAATATTACATAAATTGTGCACTTTGGGCTAAAAA//CATTTTCATTATTTTAGCCAAAAGTGCACAATTTTTTCACATAACCGTCTAACTAAAATGGAATTGGGCC

Academ-N4_ADi

DF970920.1_[520454-517482]
ACAAATTGACAAGGATAGTGTGGTCGCTTAACCCAAAATTTGAGCCGGATCATGACAAAGCGGACAAAAG//AACTTTGGCACTTTTGTCCAGTCTAACACGATCATTCCGCTAAGCGGCCAGACTAGGATGCTTATGTGAA
DF971062.1_[146057-143270]
CAAACAGTGGCTAAATAGTCTGGTCGGTTAATGCAAAATTTGAGCCGGATCGCGATAAAGCGGACAAAAG//AATTTTGTCACTTTTGTCCAGTCTGTCAGGATCATTCCGCTAAGCGACCAGACTAAAAGTATGTCTGTTG
DF971231.1_[126496-129444]
CCGTGTTTTCAATTATAGTCTGGTCGCTTAATGCAAAATTTGAGCCGGATCGTGACAAAGCGGACAAAAG//AATTTTGGCACTTTTGTCCAGTCTGTCACGATCATTCCGCTAAGCGACCAGACTATTAGTAGAAGGTTAT
DF971286.1_[17868-15028]
AAACTGTCTTGCTATTAGTCCGGTCGCTTAATGCAAAATTTGAGCCAGATCGTGACAAAGCGGACAAAAG//AATTTTGGCACTTTTGTCCAGTATCTCACGATCATTCCGCTAAGAGACCAGACTATATTGCCATTGTATT

Figure S3. Termini and TSDs of *AcademHP* families from animals. TSDs are colored in red. Positions of TEs are shown in parentheses.

Priapulus caudatus (priapulus)

AcademHP-1_PrCa

```
KQ717385.1_[15604-5998]
GAGACCTCTTATCANATGATGTCAGGCAACACAGTAAAATAGGCGCGTAGAAGTGACCGCATAGAAAATA//TATTTTCTATGCGGTCACTTCTACGCGCCTATTTTACTGTGTTGCCTGACATCATGCGGGCTCTGGTGAC
```

Saccoglossus kowalevskii (acorn worm)

AcademHP-1_SKow

```
ACQM01014202.1_[9392-482]
ATGTAGCTGGAAATGTAGCTGTGGGTCCATAGCTCTGCTTGTGTCGATCTGATGTTATAATAAACGTACT//AGTACGTTTATTATAACATCAGATCGACACAAGCAGAGCTATGGACCCACAGCTAGAGGGGACCTAGTAT
ACQM01090185.1_[9254-351]
AACCGACCGAAAAATTAGCTGTGGGTCCATAGCTCTGCTTGTGTCGATCTGATGTTATAATAAACGTACT//AGTACGTTTATTATAACATCAGATCGACACAAGCAGAGCTATGGACCCACAGCTACTGGAAATGTAGTTA
ACQM01120027.1_[9244-460]
GCATCAGGCAGCTGGTAGCTGTGGGTCCATAGCTCTGCTTGTGTCGATCTGATGTTATAATAAACGTACT//AGTACGTTTATTATAACATCAGATCGACACAAGCAGAGCTATGGACCCACAGCTAACTCTATGGCTGTCA
ACQM01122246.1_[479-9324]
CTGCTACTTTTGCGTTAGCTGTGGGTCCATAGCTCTGCTTGTGTCGATCTGATGTTATAATAAACGTACT//AGTACGTTTATTATAACATCAGATCGACACAAGCAGAGCTATGGACCCACAGCTACAAACGATAATACAT
```

```
(internally deleted derivatives)
GL011285.1_[64378-57551]
AGTGGCTGTGTTACCTAGCTGTAGGTCCATAGATCTGCTTGTGTCGATCTGATGTTATAATAAACGTACT//AGTACGTTTATTATAACATCAGATCGACACAAGCAGAGCTATGGACCCACAGGTAGCAGAGCTGTGGACC
GL012061.1_[17161-16973]
ACATACAATGATGACTAGCTATGGGCCCATAGCTCTTCTTGTGTCGGTCTTTTGTCTCTGTGCATGTATT//TATCTGAAAATAAAAACAAAATAGTGACACAAGAAGAGCTATGGACCCACAACTAAATAATCACATGATT
GL012471.1_[26952-18443]
GAATATGTCCGAACATAGCTGTGGGTCCATAGCTCTGCTTGTGTCGATCTGATATAATAAACGTACTTGA//AGTACGTTTATTATAACATCAGATCGACACAAGCAGAGCTATGGACCCACAGCTAACTCTATGGCTGTCA
GL013212.1_[1068184-1068045]
CAGTAGTCTGATATATAGCTGTGGGACCATAGCTCTGCCTGTGTCGATCTGATGGTATATCAAACGTCGT//CATACGTTCAATATATCGTCAGATCGACACAAGCAGAGCTTTGGAACCACAGCTATCTGATATATTGAAC
GL013286.1_[281495-281205]
AATCACTGCTAGTACTAGCCGTGGGTTCATATCTCTTCTTGTGTCGGTCTTTGTGGTGATATTTTAGAAA//CCATCACAAAGACTAAAGACCGACACAAGAAGAGCTATGGACCAACTTACAGCTATGTTAGTGCAAATTA
GL013464.1_[384002-383236]
AGTAATTCAATTTTGTAGCTGTGGAACCATAGCTCTGCTTGTGTCAGTCTTTAGTCTTTGTAATGGTATT//AATAACATAACCAAGACTACAGACCGACACAAGAAGAGCTATGGAATCAGAGCTATCCAAGTTGTTGTCT
GL014407.1_[120945-121830]
ATGTAGCTGGAAATGTAGCTGTGGGTCCATAGCTCTGCTTGTGTCGATCTGATGTTATAATAAACGTACT//AGTACGTTTATTATAACATCAGATCGACACAAGCAGAGCTATGGACCCACAGCTACTGGAAATGTAGTTA
GL014555.1_[406644-397370]
TTTTACCAAACGATATAGCTGTGGGTCCATAGCTCTGCTTGTGTCGATCTGATGTTATAATAAACGTACT//AGTACGTTTATTATAACATCAGATCGACACAAGCAGAGCTATGGACCCACAGCTACAAACGATAATACAT
GL015690.1_[49435-49179]
CTCTATGTACTACGTTAGCTGTGGGTCCATTGCTCTTCTTGTGTCGGTCTTTTGTCTTTGTAATGGTTTA//AATACCATTACAATAACTAAAGAGCAACACAAGAAGAGCTACGGACCCATAGCTAGCACTCTGTAAACTA
GL018532.1_[32133-31068]
AGAACATCCGGCGAGTAGCTGTGGGTCCATATCTCTTCTTGTGTCTGTATTTAGTCTTTGTGATGATATT//ACGATCACAAGGACTAAAAAAAACCGATACAAGAAGAGCTATAGACCCACACCTACCAGGTAAGTTTCTG
```

Strongylocentrotus purpuratus (purple sea urchin)

AcademHP-1_SP

```
KN918009.1_[267917-283515]
GATTTGGGCAAAACATAGCCTGACCAGACGCCGTGCGTAGCACGGCGTCTGACCTCCTCCCATACCGCCA//TTTTTGGAGCAAGCGGGGTACATCCCCGAGCTTCGCTCGGGGATGTGTATATTTAGGCTGATGTTGGATC
KN919599.1_[331545-347125]
TATTTTATCTACAGTTAGCCTGACCAGACGCCGTGCGTAGCACGGCGTCTGACCTCCTCCCATACCGCCA//TTTTTGGAGCAAGCGGGGTACATCCCCGAGCTTCGCTCGGGGATGTGTATATTTAATCTACAGTATGTAC
```

```
(internally deleted derivatives)
KN914055.1_[130122-130171]
ATGCACGTGGCCGAGTAGCCTGACCAGACGCCGTGCGTAGC--------------------------------------------------------------TTCGCTCGGGGATGTGTATATTTAGTGGCCGAGGAGCTT
KN914420.1_[21171-21975]
ATGCACTAATCAATCTAGCCTGACCAGACGCCGTGCGTAGCACGGCGTCTGACCTCCTCCCATACCGCCA//TTTTTGGAGCAAGCGGGGTACATCCCCGAGCTTCGCTCGGGGATGTGTATATTTATTATCAATCATGTGA
KN918731.1_[181769-190381]
TTGTATCTCAAAAACTAGCCTGACCCAGACCCGGCCGTAGCACGGCGTCTGACCTCCTCCCATAACCGCC//TTTGGAGCAGTCGGGGTTACATCCCGAGCTTTCGCTCGGGATGTGTAATTTGATACTCAAAACTGTGAAA
```

Table S3. Non-autonomous DNA transposons newly classified as *Academ*.

Original name	Origin	New name	Lineage
DNA8-3_PGr	*Puccinia graminis*	*AcademH-N2_PGr*	*AcademH*
DNA8-3B_PGr	*Puccinia graminis*	*AcademH-N2B_PGr*	*AcademH*
DNA9-1_PGr	*Puccinia graminis*	*AcademH-N3_PGr*	*AcademH*
DNA9-1B_PGr	*Puccinia graminis*	*AcademH-N3B_PGr*	*AcademH*
DNA9-2_PGr	*Puccinia graminis*	*AcademH-N4_PGr*	*AcademH*
DNA9-3_PGr	*Puccinia graminis*	*AcademH-N5_PGr*	*AcademH*
DNA9-4_PGr	*Puccinia graminis*	*AcademH-N6_PGr*	*AcademH*
DNA7-2_PSt	*Puccinia striiformis*	*AcademH-N9_PSt*	*AcademH*
DNA7-3_PSt	*Puccinia striiformis*	*AcademH-N10_PSt*	*AcademH*
DNA9-1_PSt	*Puccinia striiformis*	*AcademH-N11_PSt*	*AcademH*
DNA9-2_PSt	*Puccinia striiformis*	*AcademH-N12_PSt*	*AcademH*
DNA-10_PSt	*Puccinia striiformis*	*AcademH-N6_PSt*	*AcademH*
DNA-15_PSt	*Puccinia striiformis*	*AcademH-N7_PSt*	*AcademH*
DNA-27_PSt	*Puccinia striiformis*	*AcademH-N8_PSt*	*AcademH*
DNA9-1_MLP	*Melampsora larici-populina*	*AcademH-N1_MLP*	*AcademH*
DNA9-8_CGi	*Crassostrea gigas*	*AcademH-N1_CGi*	*AcademH*
DNA9-2_CGi	*Crassostrea gigas*	*AcademH-N2_CGi*	*AcademH*
DNA9-2B_CGi	*Crassostrea gigas*	*AcademH-N2B_CGi*	*AcademH*
DNA-27_CGi	*Crassostrea gigas*	*AcademH-N3_CGi*	*AcademH*
DNA-9-34_NV	*Nematostella vectensis*	*AcademH-N1_NV*	*AcademH*
DNA-9-34A_NV	*Nematostella vectensis*	*AcademH-N1A_NV*	*AcademH*
DNA-9-36_NV	*Nematostella vectensis*	*AcademH-N2_NV*	*AcademH*
DNA-9-36A_NV	*Nematostella vectensis*	*AcademH-N2A_NV*	*AcademH*
DNA-3-3_NV	*Nematostella vectensis*	*Academ-N1_NV*	*AcademX*
DNA-3-4_NV	*Nematostella vectensis*	*Academ-N2_NV*	*AcademX*
DNA-3-5_NV	*Nematostella vectensis*	*Academ-N3_NV*	*AcademX*
DNA-3-6_NV	*Nematostella vectensis*	*Academ-N4_NV*	*AcademX*
DNA-3-7_NV	*Nematostella vectensis*	*Academ-N5_NV*	*AcademX*

The number just after ‘DNA’ and before ‘-‘ in the family names indicates the length of TSDs.

Table S4. *AcademHP* remnants found in teleost.

Order	Family	Species	Accession numbers
Anabantiformes	Anabantidae	*Anabas testudineus*	LR132056.1
	Osphronemidae	*Betta splendens*	LR132012.2
Carangiformes	Carangidae	*Seriola lalandi*	NW_019525454.1
Ovalentaria incertae sedis	Pomacentridae	*Stegastes partitus*	NW_007577820.1
		Acanthochromis polyacanthus	NW_019029904.1
Perciformes	Bovichtidae	*Cottoperca gobio*	LR131930.1
Pempheriformes	Lateolabracidae	*Lateolabrax maculatus*	CP032593.1
Cypriniformes	Cyprinidae	*Labeo rohita*	QBIY01012683.1, QBIY01012664.1
